# Duplicate *dmbx1 *genes regulate progenitor cell cycle and differentiation during zebrafish midbrain and retinal development

**DOI:** 10.1186/1471-213X-10-100

**Published:** 2010-09-22

**Authors:** Loksum Wong, Cameron J Weadick, Claire Kuo, Belinda SW Chang, Vincent Tropepe

**Affiliations:** 1 Department of Cell & Systems Biology, University of Toronto, 25 Harbord Street, Toronto, ON, M5S 3G5, Canada; 2 Department of Ecology & Evolutionary Biology, University of Toronto, 25 Willcocks Street, Toronto, ON, M5S 3B2, Canada; 3 Centre for the Analysis of Genome Evolution and Function, University of Toronto, 25 Willcocks Street, Toronto, ON, M5S 3B3, Canada

## Abstract

**Background:**

The *Dmbx1 *gene is important for the development of the midbrain and hindbrain, and mouse gene targeting experiments reveal that this gene is required for mediating postnatal and adult feeding behaviours. A single *Dmbx1 *gene exists in terrestrial vertebrate genomes, while teleost genomes have at least two paralogs. We compared the loss of function of the zebrafish *dmbx1a *and *dmbx1b *genes in order to gain insight into the molecular mechanism by which *dmbx1 *regulates neurogenesis, and to begin to understand why these duplicate genes have been retained in the zebrafish genome.

**Results:**

Using gene knockdown experiments we examined the function of the *dmbx1 *gene paralogs in zebrafish, *dmbx1a *and *dmbx1b *in regulating neurogenesis in the developing retina and midbrain. Dose-dependent loss of *dmbx1a *and *dmbx1b *function causes a significant reduction in growth of the midbrain and retina that is evident between 48-72 hpf. We show that this phenotype is not due to patterning defects or persistent cell death, but rather a deficit in progenitor cell cycle exit and differentiation. Analyses of the morphant retina or anterior hindbrain indicate that paralogous function is partially diverged since loss of *dmbx1a *is more severe than loss of *dmbx1b*. Molecular evolutionary analyses of the *Dmbx1 *genes suggest that while this gene family is conservative in its evolution, there was a dramatic change in selective constraint after the duplication event that gave rise to the *dmbx1a *and *dmbx1b *gene families in teleost fish, suggestive of positive selection. Interestingly, in contrast to zebrafish *dmbx1a*, over expression of the mouse *Dmbx1 *gene does not functionally compensate for the zebrafish *dmbx1a *knockdown phenotype, while over expression of the *dmbx1b *gene only partially compensates for the *dmbx1a *knockdown phenotype.

**Conclusion:**

Our data suggest that both zebrafish *dmbx1a *and *dmbx1b *genes are retained in the fish genome due to their requirement during midbrain and retinal neurogenesis, although their function is partially diverged. At the cellular level, Dmbx1 regulates cell cycle exit and differentiation of progenitor cells. The unexpected observation of putative post-duplication positive selection of teleost Dmbx1 genes, especially *dmbx1a*, and the differences in functionality between the mouse and zebrafish genes suggests that the teleost *Dmbx1 *genes may have evolved a diverged function in the regulation of neurogenesis.

## Background

The vertebrate *diencephalon/mesencephalon homeobox 1 (dmbx1) *gene belongs to the K50 subclass of paired-like homeobox genes (related to *Goosecoid*) whose expression in the neural plate has been shown to demarcate the presumptive mesencephalic (midbrain) territory in mouse [1-6], chick [2] and zebrafish [7,8]. After neural tube formation, *Dmbx1 *is predominantly expressed in the hindbrain, posterior forebrain and midbrain. Loss of function studies highlight a role for *Dmbx1 *(mouse) and *dmbx1a *(zebrafish) in proper midbrain and hindbrain development and in the case of zebrafish development of the retinotectal pathway [8,9], although in mouse the loss of function embryonic phenotype is comparatively mild. Moreover, early postnatal lethality occurs in the vast majority of *Dmbx1 *knockout mice. Those that survive to adulthood predominantly display hypophagia and hyperactivity, likely a result of defects in the development of neural circuitry involved in energy homeostasis [9,10].

Genomic sequence analyses indicate that basal metazoans, such as Ctenophores [11], and Poriferans [12], do not have a *Dmbx1 *gene. However, a putative *Dmbx1 *ortholog has been identified in the Cnidarian genome [13], suggesting a pre-bilaterian origin of the *Dmbx1 *homeobox gene family during animal evolution. Non-chordate invertebrate *Dmbx1 *orthologs have not been clearly identified, but based on overall class homology and function, the *Drosophila Pph13/Mu *gene is a plausible candidate [14]. A single *Dmbx1 *gene exists in the genomes of terrestrial vertebrates, while teleost genomes contain at least two paralogs: *dmbx1a *and *dmbx1b *(previously annotated as *mbx1 *and *mbx2*) [7], coincident with the more recent additional round of gene/genome duplication that is speculated to have occurred in the teleost lineage [15,16]. This has lead us to examine why both copies of the *dmbx1 *gene duplicate have been retained in the zebrafish genome.

The retention of functional gene duplicates (paralogs) in genomes is often attributed to their role in buffering against loss-of-function mutations in one copy of an essential gene [17,18]. However, the proportion of genes that when deleted are embryonic lethal or lead to infertility in mouse (i.e. essential in a laboratory context) is not significantly different between duplicates and singletons [19,20]. Even though protein sequences of some paralogous genes in vertebrates are functionally interchangeable [21,22], paralogs can evolve distinct expression patterns and functions. The subfunctionalization model suggests that subsequent to gene duplication, degenerative mutations in regulatory or coding regions of the gene result in complementary expression patterns or function, respectively, the composite of which would be representative of the pre-duplicated ancestral gene [23,24]. This appears to be a predominant mechanism in teleosts [22,25-28]. Neofunctionalization has also been proposed to account for the retention of duplicate genes [29]. A recent genome wide study in *Xenopus laevis *indicated that as many as 6% of duplicate genes exhibit an asymmetric rate of non-synonymous substitution in one of the paralogs, which is consistent with a neofunctionalization model for paralog retention [30]. However, experimental evidence for neofunctionalization in vertebrates is limited.

Our previous gene expression analyses of zebrafish *dmbx1a *and *dmbx1b *showed that despite conservation in the regulation of expression within particular regions (e.g. midbrain primordium), there were clear differences in onset, spatial distribution and relative abundance during the first two days of development [7]. These differences in regulation correlated with variation in the extent of genomic sequence conservation between the paralogs in three separate fish species [7]. We also noted that although the amino acid sequence similarity was high (72%) between the paralogs, this was mostly due to the N-terminus and DNA binding domain, whereas much of the C-terminus contained relatively divergent sequence. From these results, we concluded that the zebrafish *dmbx1 *paralogs might have been subfunctionalized during the course of their evolution.

Here, we examined the functional requirement of zebrafish *dmbx1a *and *dmbx1b *during neural development using morpholino oligonucleotide based loss of function to decipher a possible mechanism for the retention of these duplicate genes. We demonstrate that neither functional redundancy nor subfunctionalization is an adequate model to account for the retention of these paralogs in the zebrafish genome. Instead, we show that both genes are required during development. Loss of function of *dmbx1a *and *dmbx1b *causes a prolonged progenitor cell cycle in the midbrain and retina, which results in reduced growth, and a significant defect in differentiation. Although these knockdown phenotypes can be rescued by exogenous zebrafish *dmbx1 *mRNA, they cannot be efficiently rescued by exogenous mouse *Dmbx1 *mRNA to the same degree. Finally, analyses of the rate of non-synonymous to synonymous substitution in the coding region of *Dmbx1 *genes provide evidence for post-duplication positive selection in the *dmbx1a *and *dmbx1b *gene families of teleost fish. These data suggest that both zebrafish *dmbx1 *paralogous genes are non-redundantly required for cell cycle exit regulation and differentiation. Although the differences in gene expression domains between the *dmbx1 *paralogs is subtle at late embryonic and early larval stages, the knockdown data demonstrate that regional functional specialization, especially with respect to *dmbx1a*, exists. Thus, the function of *dmbx1 *genes in teleost fish may be evolutionarily diverged.

## Results

### Discrete variation in the timing and distribution of *dmbx1a *and *dmbx1b *gene expression during the early larval stage

Previous analyses suggested that the *dmbx1a *and *dmbx1b *paralogs displayed partially divergent spatial and temporal patterns of gene expression in early development [7]. For example, between shield and tailbud stages, *dmbx1a *has a characteristic annulus expression pattern specifying the midbrain territory, as well as progenitor cells of retinal and diencephalic lineages, whereas *dmbx1b *expression during these stages is barely detectable and appears later at mid-somitogenesis only in the presumptive midbrain [7,8]. Between 24 - 48 hpf, the expression domains of these two genes partially overlapped in the midbrain and hindbrain, but neither gene is expressed in the retina. We analyzed the expression patterns of the *dmbx1 *paralogs in the brain and retina during the late embryonic to early larval stages using in situ hybridization in order to clarify where the paralogs exert their function during development.

**Figure 1:**
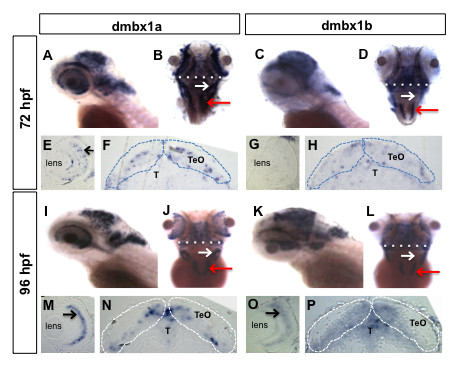
**Spatiotemporal expression patterns of *dmbx1a *and *dmbx1b *at 72 hpf and 96 hpf**. Lateral view, anterior to the left (A, C, I, K); dorsal view, anterior to the top (B, D, J, L). Coronal plastic sections (~1 μm; dorsal to the top) of the retina (E, G, M, O) and midbrain (F, H, N, P) were obtained after whole mount labeling with RNA probes. White dotted line in B and D define the position of the representative coronal plastic section shown in F and H, respectively. White dotted line in J and L define the position of the representative coronal plastic section shown in N and P, respectively. White arrow in B, D, J, L demarcates the rostrolateral region of the hindbrain (where the EG is located), and red arrow demarcates the expression in the medulla oblongata. Black arrows in E, M and O point to the INL (inner nuclear layer). The optic tectum (TeO) is demarcated by dotted lines in F, H, N, P and the region of the tegmentum (T) lies just ventral to the TeO. The position of the lens is denoted in E, G, M, O. hpf, hours post-fertilization.

At 72 hpf, *dmbx1a *was expressed in the optic tectum (TeO) and very weakly in the tegmentum (T) (Figure 1A, B, F). Scattered cells of the retinal inner nuclear layer (INL) and ganglion cell layer (GCL) also express *dmbx1a *(Figure 1E). However, there is very weak or no *dmbx1a *expression in the photoreceptor layer and no expression in the ciliary marginal zone (CMZ), where the post-embryonic retinal stem and progenitor cells undergo continual proliferation [31]. *Dmbx1a *is also expressed in discrete hindbrain cell populations that appear to demarcate the cerebellar eminentia granularis (EG) anteriorly (Figure 1A; B, white arrow) and the medulla oblongata posteriorly (Figures 1A; B, red arrow). In addition, *dmbx1b *was expressed throughout the midbrain and within the medulla at 72 hpf (Figure 1C; D, red arrow), but only very weakly in the INL and EG (Figures 1D, G).

Expression analyses at 96 hpf continued to reveal discrete variation in the distribution of cells expressing *dmbx1a *and *dmbx1b*. For example, *dmbx1a *is predominantly localized to the medial and lateral compartments of the TeO in the midbrain and weak expression is detected in the tegmentum (Figure 1I,J,N), whereas *dmbx1b *expression extends more ventrally to encompass more of the tegmentum (Figure 1K,L,P). This difference in dorsoventral expression domains is consistent with our previous observations at 24 - 48 hpf [7]. Furthermore, the predominantly tectal expression of *dmbx1a *is similar to that of mouse *dmbx1 *[4]. Expression of *dmbx1b *in the anterolateral hindbrain is also more substantial at this stage, but not quite comparable to *dmbx1a *(Figure 1I,K). Finally, both *dmbx1a *and *dmbx1b *are expressed throughout the retinal INL, but *dmbx1a *appears enhanced and localized to the central region of the INL, whereas *dmbx1b *expression is relatively diffuse and less intense throughout most layers (Figure 1M,O).

Taking into account the relative temporal, spatial, and quantitative expression patterns of the *dmbx1 *paralogs in the first 4 days of life ([7]; present results), our data suggests that *dmbx1 *genes would predominantly function in midbrain formation, plus a role in retinal and hindbrain development during later differentiation. Therefore, we sought to compare the functional requirement of Dmbx1a and Dmbx1b.

### Gene knockdown using *dmbx1 *paralog specific antisense morpholino oligonucleotides

**Figure 2:**
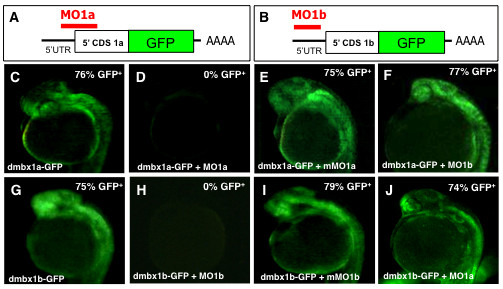
**Specificity of morpholino induced *dmbx1a *and *dmbx1b *knockdown using fusion protein constructs Dmbx1a-GFP and Dmbx1b-GFP**. (A, B) Schematic diagrams showing sequence of fusion constructs: 5' UTR (black horizontal line); 5' end of the CDS for either *dmbx1a *or *dmbx1b *(white box), the full-length coding sequence for GFP (green box); site of polyadenylation (AAAA); relative position binding sites (red horizontal line) where the MOs block translation of the fusion proteins. (C-J) *dmbx1a*- and *dmbx1b*-GFP mRNA was either injected alone or co-injected with MOs as indicated at the bottom left of each panel, and embryos were examined for the presence of GFP fluorescence at 24 hpf (lateral view with anterior to the left). The percentage of GFP positive embryos is shown on the top right. UTR, untranslated region; CDS, coding sequence; MO, morpholino; mMO, mismatch morpholino; GFP, green fluorescent protein; hpf, hours post-fertilization.

Given the high degree of sequence similarity between the *dmbx1 *paralogs, our strategy for using antisense morpholino oligonucleotide (MO) based knockdown required confirmation that paralog specific MOs could independently block translation of *dmbx1a *and *dmbx1b *mRNA. In the absence of commercially available antibodies that are validated to detect zebrafish Dmbx1a or Dmbx1b proteins, we opted for an alternative approach to estimate the level of protein knockdown for each paralog. We constructed in-frame GFP fusion constructs containing the unique paralog specific MO targeting sequences, and co-injected each of the MOs with their corresponding *in vitro *transcribed fusion mRNAs (Figure 2A,B). When either *dmbx1a-GFP *(n = 62) or *dmbx1b-GFP *(n = 73) fusion mRNA was injected, ~75% of the embryos had bright ubiquitous GFP protein expression after 24 hpf (Figure 2C,G). GFP expression was completely suppressed in embryos that were co-injected with MO1a + *dmbx1a-GFP *(Figure 2D, n = 74) or MO1b + *dmbx1b-GFP *(Figure 2H, n = 59), indicating that the MOs result in very efficient translation inhibition.

In order to control for MO sequence specificity, we also co-injected the GFP fusion mRNA constructs with a 5-bp mismatched MO (mMO1a or mMO1b; see Materials & Methods for details) and quantified the percentage of injected embryos that were GFP positive after 24 hpf. Of the embryos that were co-injected with mMO1a + *dmbx1a-GFP *(Figure 2E, n = 53) or mMO1b + *dmbx1b-GFP *(Figure 2I, n = 64), ~75 - 80% of the embryos demonstrated ubiquitous GFP expression, which was similar to the percentage of fusion construct injected embryos expressing GFP without MO co-injection. These results indicate that the knockdown of either GFP fusion construct depends precisely on the complementary MO sequences.

We further examined the paralog specific knockdown characteristics of the MOs in order to confirm that there were no cross-target effects. Co-injection of MO1b + *dmbx1a-GFP *(Figure 2F, n = 60) or MO1a + *dmbx1b-GFP *(Figure 2J, n = 62) resulted in ~75 - 80% of the embryos with ubiquitous GFP expression in injected embryos after 24 hpf. Again, the numbers of GFP positive embryos within the test pool were comparable to the results obtained from injecting the GFP fusion mRNA alone, indicating that it is unlikely that cross targeting of MOs is occurring. These data suggest that the MO knockdown of the Dmbx1-GFP fusion proteins, and by inference the endogenous Dmbx1 proteins, is both efficient and paralog specific.

### Midbrain growth defects in embryos with reduced levels of either Dmbx1a or Dmbx1b

**Figure 3:**
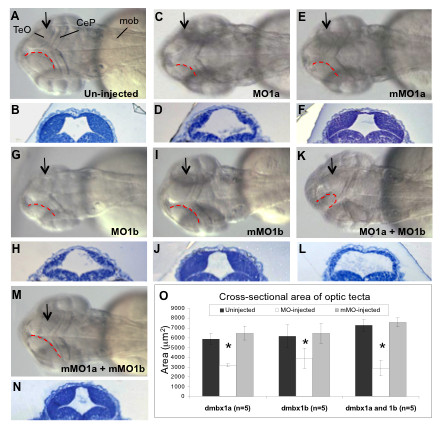
**Hypoplasia of the optic tectum in *dmbx1 *morphants at 48 hpf**. Gross morphologies of the optic tecta were compared between morphants (C, G, K) and both the un-injected (A) and mMO-injected (E, I, M) embryos. Dorsal view, anterior to the left. Contour of the medial-posterior ridge of the optic tectum is demarcated by red doted lines. The MOs used in each group are shown on the bottom right of the panel. Arrows in A, C, E, G, I, K, M represent the relative position where ~ 1 μm plastic sections were obtained as depicted in B, D, F, H, J, L, N, respectively. Measurements of the cross-sectional tectal area are summarized in the graph (O). Asterisk indicates significant difference (p < 0.05) between the morphant samples and the controls. TeO, optic tectum; CeP, cerebellar plate; mob, medulla oblongata; MO, morpholino; mMO, mismatch morpholino.

The early onset and sustained expression of *dmbx1a *and *dmbx1b *within the midbrain suggested that these genes play an important role in the development of this neuroanatomical structure. Morphological analyses were carried out to compare the MO-injected embryos (10 ng/embryo each) with mMO-injected (10 ng/embryo each) as well as un-injected controls. After 24 hpf, we were able to detect subtle differences in the size of the midbrain (data not shown), but this morphological change was more prominent at 48 hpf, where the size of the dorsal tectum of the MO1a-injected embryos was reduced (Figure 3C,D) compared to un-injected (Figure 3A,B) and mMO1a-injected embryos (Figure 3E,F), consistent with previous results using the same morpholino (MO1a) [8]. We observed that the overall cross-sectional area (or thickness) of the tectal hemispheres is diminished in the MO1a morphant, but that the shape of the contours of the tectal hemispheres and the extent of the cerebellar plate (CeP) remains relatively unaffected. We analyzed transverse sections to quantify these differences by measuring the average cross-sectional area of the tectal wall unilaterally [from the lateral sulcus separating the tectum dorsally from the region of the torus semicircularis (TS) ventrally] and observed a reduction of ~ 50% in the MO1a morphants compared to controls at the same position along the anteroposterior axis (Figure 3O). Interestingly, the tectal morphology of MO1b morphant embryos (Figure 3G,H) was also affected compared to mMO1b control injected embryos (Figure 3I,J), and cross-sectional area measurements revealed a ~ 35% reduction in size (Figure 3O).

Knockdown of both *dmbx1 *genes simultaneously (MO1a + MO1b) resulted in an obvious change in the overall morphology of the tectum (Figure 3K,L) compared to the double control injected (mMO1a + mMO1b) embryos (Figure 3M). In order to compare with the single morphant embryos, we co-injected 5 ng of each morpholino. In contrast to the single morphant phenotype, we observed that the shape of the tectal hemispheres in the double morphant often was abnormal and that the extent of the CeP was reduced, which can be observed from a dorsal perspective (compare red dotted line in Figure 3K with red dotted lines in 3A, C, G). Despite the more extensive morphological alterations in the double morphants, the overall cross-sectional area of the tectal hemispheres was reduced to a similar degree (~ 60%) when compared to the differences observed in the single morphant analyses (Figure 3L,O). Overall we observed that the average tectal cross-sectional areas in a transverse section of the un-injected and mMO1 injected embryos ranged between 5800 - 7600 μm^2^, whereas the morphant embryos had an average area that ranged between 2800-3900 μm^2^, which were statistically significantly less than their cognate controls, but not significantly different from each other. The overall growth of the morphant embryos was not significantly impaired by 48 hpf [length WT = 2986 ± 51 μm; MO1a+b = 2910 ± 210 μm; mMO1a+b = 3018 ± 65 μm (n = 5 per group)] and we confirmed that after normalizing the tectal cross-sectional area measurements to embryo length, the area in the double morphants was significantly reduced compared to un-injected (t = 9.5, p = 1.2 × 10^-5^; n = 5 per group) as well as the mMO1a + mMO1b injected (t = 11.2, p = 3.0 × 10^-5^; n = 5 per group) embryos, whereas the two control groups were not significantly different (t = 0.7, p = 0.5; n = 5 per group).

### Midbrain gene expression defects in embryos with reduced levels of either Dmbx1a or Dmbx1b

In order to determine whether the loss of function of *dmbx1a *and *dmbx1b *altered neural differentiation, various neural markers were examined using whole-mount *in situ *hybridization focusing on the combined knockdown of *dmbx1 *genes. *Otx2*, *foxb1.2*, and *lim1 *were examined at 48 hpf and the expression of these markers in the optic tecta was decreased in the morphants compared to control embryos (Additional file 1). The expression of *otx2 *in the retina indicated a reduced overall size in the morphant embryos beginning at 48 hpf (Additional file 1A - C). Expression of *foxb1.2 *in the tectum was virtually eliminated in the double morphants (Additional file 1D - F), but there was no change in *foxb1.2 *expression in the ventral diencephalic domain or in the MHB. Similarly, expression of *lim1 *(Additional file 1G - I) was significantly reduced in the posterior tectum adjacent to the MHB, but not altered in the cerebellar primordium on the posterior side of the MHB. Expression of *egr2b (krox20) *(Additional file 1J - L) and *pax2a *(Additional file 1M - O) confirmed that early specification and segmentation in the hindbrain region was relatively unaffected in the double morphant embryos at this stage. Nonetheless, judging by the *foxb1.2 *expression, specific subpopulations of hindbrain precursors may be affected and analyses of transverse sections demonstrate that overall tissue growth of the hindbrain was reduced (data not shown). Interestingly, *pax2a *expression within the optic stalk region was significantly enhanced (Additional file 1P - R). This is consistent with the fact that *dmbx1a *expression during gastrula stages partially overlaps with anterior neural plate cells destined for a retinal fate [7,8] and suggests that in the absence of *dmbx1a *some of these cells are transformed toward an optic stalk identity [32,33] while delaying further retinal development during optic cup formation. This may partly explain the persistent *rx1 *progenitor cell marker expression in the *dmbx1a *morphant retina up to 48 hpf [8], even though *dmbx1 *genes are not apparently expressed in the retina proper before this stage. Finally, we examined in more detail the expression of markers of the MHB (*eng3*, *fgf8*, *erm*, *pax2a*, *wnt1*), as well as markers for various telencephalic and diencephalic structures (*shh, dlx2a, axial, emx2*) between 24 - 48 hpf. In general, we observed no significant differences between the double morphant embryos and controls in the MHB (Additional file 2) or the forebrain (data not shown), although we note that there is an apparent increase in *fgf8 *expression in rostral telencephalon of the morphant embryos (Additional file 2D-F), which correlates with a loss of *dlx2a *in the ventral telencephalon (data not shown). This could have implications for telencephalic development.

**Figure 4:**
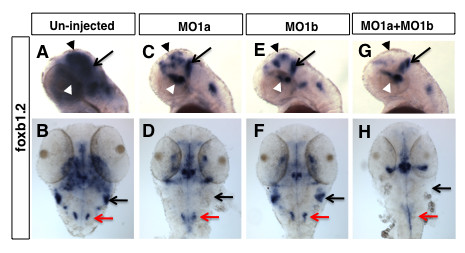
**Distinct patterns of hindbrain *foxb1.2 *expression in *dmbx1a *and *dmbx1b *morphant embryos**. Lateral view anterior to the left (A, C, E, G) and dorsal view anterior to the top (B, D, F, H) of 48 hpf embryos un-injected (A, B), or injected with MO1a (C, D), MO1b (E, F) or MO1a + MO1b (G, H). The mismatch control injected morpholinos resembled the un-injected controls and are not shown. (A, C, E, G) Black arrow indicates the position of the MHB, white arrowhead demarcates the position of the ventral diencephalic/hypothalamic region of the forebrain, and black arrowhead demarcate expression domain in the region of optic tectum. Black arrow in B, D, F, H demarcates the region of the rostrolateral hindbrain (where EG is located), and red arrow demarcates the expression in the medulla oblongata. MO, morpholino; MHB, midbrain-hindbrain boundary; hpf, hours post-fertilization

The specific loss of *foxb1.2 *expression in midbrain and hindbrain regions in the double morphants suggested that the development of specific sub-regions with *dmbx1 *gene expression were compromised as a result of gene knock down. However, given that *dmbx1a *and *dmbx1b *have partially non-overlapping expression domains, we also investigated *foxb1.2 *expression in single morphant embryos. Knockdown of either Dmbx1a or Dmbx1b caused a significant reduction in *foxb1.2 *expression in the midbrain, which was enhanced further in the double morphants (Figure 4A,C,E,G, black arrow head). However, in the hindbrain, *foxb1.2 *expression in the anterolateral domain (presumptive EG) was eliminated only in the presence of MO1a and not MO1b (black arrow in Figure 4B,D,F,H), which is consistent with the expression of *dmbx1a *and absence of *dmbx1b *in this region at 48 hpf (Figure 1). Although the trigeminal ganglion is also present in this anterior-lateral domain, it does not appear to be defective in the MO1a morphant as indicated by the relatively normal expression of GFP in the *isl2b:GFP *transgenic embryos (see Additional file 4B,D,F,H). The posterior-medial domain of *foxb1.2 *expression was reduced in the MO1a + MO1b injected embryos (red arrow in Figure 4B,D,F,H). We performed a dose-response analysis for morpholino efficacy using *foxb1.2 *gene expression as a reliable correlated readout for the midbrain development defects observed at 48 hpf. When 5 ng of either MO1a or MO1b was used, there was no discernable change in *foxb1.2 *expression in the midbrain (or hindbrain) compared to un-injected controls (Additional file 3). However, when 10 ng of either MO1a or MO1b was injected separately, a similar reduction in *foxb1.2 *expression and size of the midbrain was observed (Figure 3; Additional file 3). Combined injection of 5 ng MO1a and 5 ng MO1b caused a significant reduction in *foxb1.2 *expression (Additional file 3). Furthermore, we confirmed that the ventral diencephalic/hypothalamic (white arrowhead Figure 4A,C,E,G) and MHB (black arrow Figure 4A,C,E,G) expression of *foxb1.2 *were relatively less affected in the morphants. Thus, *dmbx1a *and *dmbx1b *appear to be independently required for region-specific midbrain and hindbrain development perhaps through a synergistic mechanism.

### Retinal growth defects in embryos with reduced levels of either Dmbx1a or Dmbx1b

Comparing our previous expression analyses [7] with the present results (Figure 1), the onset of expression of *dmbx1a *within the retina (after the optic cup is formed) occurs ~48 hpf, whereas retinal expression of *dmbx1b *appears to be similar but slightly delayed. Thus, we reasoned that a requirement for both *dmbx1 *paralogs in the retina proper would be manifest most prominently between 72 - 96 hpf. Indeed, we did not observe any significant gross morphological defects in the retina in combined MO1a + MO1b injected embryos compared to controls at 24 hpf (data not shown).

**Figure 5:**
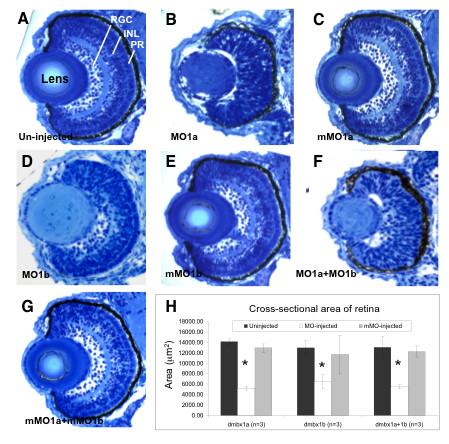
**Retinal hypoplasia in *dmbx1 *morphants at 72 hpf**. Coronal plastic sections (~ 1 μm) of retina from un-injected (A), MO-injected (B, D, F), and mMO-injected (C, E, G) embryos. Layers in the retina can be clearly distinguishable at this stage: retinal ganglion cell layer (RGC), inner nuclear layer (INL), and photoreceptor layer (PR). Measurements of the retinal cross-sectional area summarized in the graph (H). Asterisk indicates significant difference (p < 0.05) between the morphant samples and the controls. MO, morpholino; mMO, mismatch morpholino.

In order to determine the relative requirement of *dmbx1a *and *dmbx1b *in the development of the retina, we examined transverse sections of the retina at 72 hpf. Single MO1a injected embryos had severely compromised differentiation and lamination (Figure 5B) compared to the un-injected (Figure 5A) or mMO1a injected (Figure 5C) embryos. Furthermore, there was a significant decrease in the average overall area of a mid-transverse section (thickness) of the retina in the *dmbx1a *morphants compared to controls (Figure 5H). In contrast, the MO1b injected embryos displayed a relatively mild retinal differentiation phenotype (most prominently in the dorsal regions) (Figure 5D) compared to the un-injected (Figure 5A) and mMO1b injected (Figure 5E) controls. However, similar to the *dmbx1a *morphants, the *dmbx1b *morphants demonstrated an overall reduction in the average mid-transverse area of the retina (Figure 5H). From these results we predicted that the combined MO1a + MO1b injected embryos would resemble the MO1a injected embryos. Indeed, the severe defect in differentiation in the double morphant (Figure 5F) compared to controls (Figure 5A,G) appeared identical to that of the single MO1a morphant. Interestingly, the reduction in the average mid-transverse area in the double morphants was not significantly different from that observed in the single morphant embryos, ranging from 40 - 60% less than controls (Figure 5H). These observations indicate that retinal growth is primarily dependent on the *dmbx1a *paralog, which can partially compensate for the lack of *dmbx1b*, but that *dmbx1b *is also required for the proper continued growth of the retina (from approximately 48 - 72 hpf).

The overall growth by 72 hpf [length WT = 3302 ± 54 μm; MO1a+b = 2996 ± 159 μm; mMO1a+b = 3278 ± 65 μm (n = 5 per group)] of the double morphant embryos was significantly less than un-injected (t = 3.36, p = 0.01; n = 5 per group) and mMO1a + mMO1b injected (t = 3.36, p = 0.01; n = 5 per group) embryos. However, despite this difference, we still confirmed that after normalizing the retinal area measurements to embryo length, the area in the double morphants was significantly reduced compared to un-injected (t = 7.7, p = 2.4 × 10^-4^; n = 5 per group) as well as the mMO1a + mMO1b injected (t = 10.0, p = 2.1 × 10^-5^; n = 5 per group) embryos, whereas the two control groups were not significantly different (t = 0.08, p = 0.94; n = 5 per group). The overall length difference in the morphants is directly related to the reduced midbrain, retina and hindbrain since other regions of the embryo appeared normal in size (data not shown). We also observed that the lens was smaller in MO1a and MO1a+MO1b morphants. Although the peripherally localized epithelial cells are present, in all cases there appears to be a defect in the differentiation of fiber cells forming the characteristic darkly stained concentric rings. This defect may be due to a secondary disruption in the normal lens-retina interactions during development. However, we did not explore this possibility and further experiments are required to characterize this lens defect in more detail.

### Retinal differentiation defects in embryos with reduced levels of either Dmbx1a or Dmbx1b

**Figure 6:**
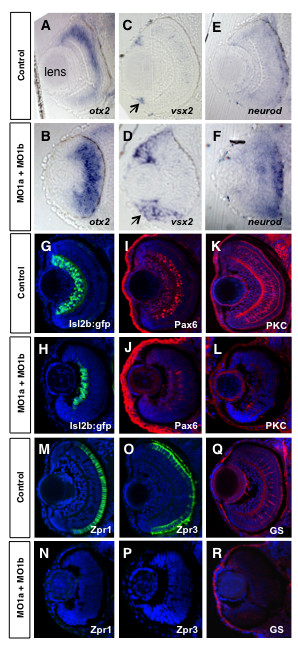
**Altered retinal gene expression in morphant embryos**. Coronal plastic sections (~ 1 μm) of retina at 72 hpf after whole mount in situ hybridization labeling for progenitor markers from control mMO1a + mMO1b injected (A, C, E), or MO1a + MO1b injected (B, D, F) embryos. Position of the lens (shown in A) is similar for all panels. Arrows in C and D demarcate the ciliary marginal zone (CMZ). Cryosections (~15 μm) of control (G, I, K, M, O, Q) and MO1a + MO1b (H, J, L, N, P, R) embryos immunolabelled with antibodies for indicated differentiation markers (red or green) and counter-stained with DAPI (blue). MO, morpholino.

In order to gain more insight into retinal differentiation changes due to reduced levels of the *dmbx1 *genes, we examined a panel of markers on transverse sections using *in situ *hybridization and immunohistochemistry in double morphant embryos at 72 hpf. In control embryos, *otx2 *was mostly absent from the RGC layer, ONL and the CMZ, but expressed in central regions of the INL at 72 hpf (Figure 6A). In the double morphant embryos, *otx2 *expression appeared expanded and relatively uniform throughout the central retina, but not in the CMZ (Figure 6B). Consistent with the notion that differentiation of at least some of the retinal cell types is reduced in the morphant embryos, we observed a significant expansion of *vsx2*-expressing stem and/or progenitor cells in the CMZ [34] compared to controls (Figure 6C,D). There was also an increase in *neurod*-expressing cells (Figure 6E,F), a marker of photoreceptor progenitor cells [35]. Together these data reveal that reduced levels of Dmbx1 results in a persistent progenitor identity in cells throughout the retina, which correlates with our histological analyses.

To gain greater insight into the differentiation defects of the morphant embryos, we analyzed several retinal cell-type specific markers using immunohistochemistry and confocal microscopy. Using an *isl2b:gfp *transgene to mark retinal ganglion cells [36], we observed a reduction, but not a complete loss, of retinal ganglion cells (GFP+) in the double morphant embryos compared to controls (Figure 6G,H). The expression of Pax6, which marks most amacrine cells and a subpopulation of ganglion cells [34], was also reduced (Figure 6I,J). Consistent with these observations, expression of PKC, which marks bipolar neurons, general markers for cone photoreceptors (Zpr1), rod photoreceptors (Zpr3), and Müller glia (glutamine synthetase, GS) were almost completely abolished in the double morphant embryos (Figure 6K-R). Thus, although there are some differentiated ganglion cells and amacrine cells, most cells in the *dmbx1 *double morphant retina have not differentiated by 72 hpf, and this correlates with the relative increase in progenitor cell marker expression and morphology.

Based on our histological analyses, we predicted that the effects of gene knockdown on retinal cell differentiation might be paralog-specific. Thus, we carefully examined the expression of *rhodopsin *(*rho*) in single morphant embryos compared to the double morphants as a marker for photoreceptor differentiation. Consistent with our histological and immunolabeling data, MO1a injected embryos (Additional file 4C) showed a significant loss of *rho *expression that mimicked the double morphant phenotype (Additional file 4G). The MO1b injected embryos, in contrast, had a less attenuated *rho *expression phenotype (Additional file 4E), confirming that loss of *dmbx1b *in the retina results in a less severe differentiation phenotype. Previous analyses demonstrated that knockdown of *dmbx1a *alone caused defects in retinotectal projections and reduced terminal fields within the TeO [8]. We confirmed this observation in MO1a injected embryos using a *Tg(isl2b:GFP)^zc7 ^*transgenic zebrafish line that robustly marks the RGCs in the retina, their axonal trajectories along the retinotectal pathway, and the contralateral terminal fields in the TeO [36]. Approximately 75% of MO1a injected embryos (n = 25; Additional file 4D) showed a defasciculated optic nerve prior to the chiasma compared to control (n = 30) (white arrowhead, Additional file 4B) as well as reduced terminal fields in the TeO (white arrow, Additional file 4D). In contrast, the retinotectal projection in MO1b injected embryos (n = 25) appeared normal (Additional file 4F), which could be due to the presence of normal levels of *dmbx1a*. If so, then a functional role of *dmbx1b *in RGC development may be absent. Thus, we predicted that the retinotectal projection in the double morphants would resemble that of the MO1a injected embryos. Again in approximately 75% of embryos, we observed a significant defect in the retinotectal projections when both paralogs are knocked down (n = 35), with significantly reduced terminal fields in the TeO and optic nerve defasciculation (Additional file 4H). These data suggest that *dmbx1a *has a predominant role in the development of the retinotectal projection, compared to *dmbx1b*, which was due to a defect in RGC differentiation, a defect in the development of the TeO, or both.

We examined the relative requirement of each paralog in retinal differentiation by performing a similar dose response analysis as mentioned above using *rho *gene expression as a reliable correlated readout for the retinal defects observed at 72 hpf. In contrast to the observations made for *foxb1.2 *expression in the midbrain/hindbrain, 5 ng of MO1a resulted in a substantial reduction (~50%) in the extent of *rho *expression in the retina, whereas 5 ng of MO1b resulted in a negligible difference in *rho *expression compared to un-injected controls (Additional file 3). However, when 5 ng of MO1a and MO1b was combined for a total of 10 ng injected, we observed a significant reduction in *rho *expression (Additional file 3). When 10 ng of MO1a was injected individually, a similar reduction in *rho *expression and size of the retina is observed when compared to the double morphants derived from injections of 5 ng of each morpholino combined (Additional file 3). Unexpectedly, when 10 ng of MO1b was injected individually, a relatively mild reduction in *rho *expression was observed compared to controls (Additional file 3). These data suggest that *dmbx1a *has a predominant functional role in retinal differentiation and that *dmbx1b *may only have a minor, additive role.

### Defects in tissue size and cell differentiation in the midbrain and retina are not due to persistent cell death

Our data comparing the *dmbx1 *single and double morphants demonstrate that the size of the midbrain and retina are reduced and that neural differentiation is significantly attenuated. One possible mechanism to account for this phenotype is increased cell death in progenitor cells within these regions, resulting in diminished growth and differentiation potential of the tissue. We tested this hypothesis by examining the level of cell death at 24, 48 and 72 hpf using the chromatin binding fluorescent marker acridine orange (AO) to detect apoptotic cells in live embryos [37]. An increased number of AO+ cells was observed in the midbrain and hindbrain at 24 hpf in MO1a + MO1b injected embryos compared to un-injected and mMO1a + mMO1b injected embryos (Additional file 5A, B). However, the growth defects in morphant embryos were not apparent until after this stage. Thus, to test whether persistent apoptotic cell death was the cause of the growth defects, we analyzed AO labelling at 48 and 72 hpf. Little or no apoptosis was detected in any of the groups at 48 hpf (Additional file 5C, D; data not shown). At 72 hpf, there is substantial remodelling occurring within the teleostean retinotectal pathway, which results in apoptotic cell death in both retinal and tectal tissue [38]. This was detected in un-injected embryos and similar levels of cell death were observed in embryos injected with a combination of mMO1a + mMO1b (Additional file 5E, K). In contrast, combined MO1a + MO1b injected embryos showed less AO labelling in both the midbrain and the retina compared to controls (Additional file 5F, L). We confirmed these observations by staining for activated Caspase3 and by performing TUNEL labelling at 72 hpf to detect apoptotic cells. The number of Caspase3+ or TUNEL+ nuclei in transverse sections of the midbrain (Additional file 5G-J) or retina (Additional file 5M-P) at this stage were not different between the controls and double morphants. Furthermore, examination of our semi-thin plastic sections from morphant tissues did not reveal any evidence for enhanced pyknosis, cellular debris or large autophagic vacuoles relative to controls at any time point between 48 - 96 hpf, suggesting that neither necrosis nor autophagy appears to account for the morphant phenotype. Thus, these data indicate that persistent cell death likely does not account for the defects in size and differentiation of the midbrain and retina in *dmbx1 *morphants.

### Dmbx1a and Dmbx1b are required for normal progenitor cell cycle regulation

**Figure 7:**
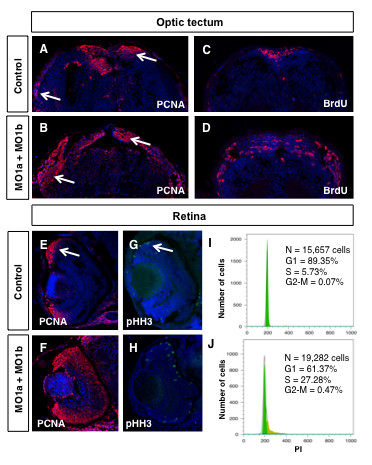
**Proliferation defects in *dmbx1 *morphant embryos**. Embryos at 72 hpf were examined for the presence of proliferating cells using immunohistochemistry with antibodies to PCNA, pHH3 or BrdU (30 min survival). Coronal (~15 μm) sections of midbrain (A-D, dorsal to the top) and retina (E-H, dorsal to the top, lens to the left). Arrow in A and B demarcates the proliferative zones of the dorsal and lateral TeO. Arrow in E and G demarcates the CMZ. (I, J) Flow cytometric analysis of retinal cells derived from un-injected or MO1a + MO1b injected embryos at 72 hpf using propidium iodide labeling. N, total number of cells analyzed; G1, gap-1 phase of the cell cycle; S, synthesis phase of the cell cycle; G2, gap-2 phase of the cell cycle; MO, morpholino; mMO, mismatch morpholino; hpf, hours post-fertilization.

An alternative mechanism that can account for the reduced size and attenuated differentiation of midbrain and retinal tissue may entail changes in the capacity for progenitor cell proliferation. We analyzed nuclear PCNA protein expression by immunohistochemistry to label cells that are actively in cell cycle [39]. By 72 hpf cell proliferation becomes substantially restricted to periventricular locations within the brain [39], and the CMZ in the retina [40] under normal conditions. Thus, we predicted that a defect in cell cycle regulation would be most evident by 72 hpf when neurogenic compartments are normally relatively small and very well circumscribed. PCNA labelling in the brain at 72 hpf revealed that cell proliferation in the optic tectum is maintained throughout the entire perimeter bilaterally in the double morphants (Figure 7B), whereas at this stage in control embryos the tectal neurogenic zones are more restricted to the lateral (bordering with the torus semicircularis anlagen) and most prominently the medial (bordering with the torus longitudinalis anlagen) compartments (Figure 7A). These observations reveal that the reduction of Dmbx1a and Dmbx1b expression caused an increase in the number of actively cycling cells in the midbrain.

To confirm these results with another cell cycle marker, we tested whether cells can incorporate 5-bromo-2'-deoxyuridine (BrdU), a thymidine analog, during S phase of the cell cycle. Embryos at 72 hpf received a single intracerebroventricular bolus of 5 mM BrdU dissolved in embryo media, and subsequently processed for immunohistochemistry after a 30 min survival period. The combined knockdown of Dmbx1a and Dmbx1b caused a significant increase in BrdU labelled cells (Figure 7D), compared to controls (Figure 7C).

**Figure 8:**
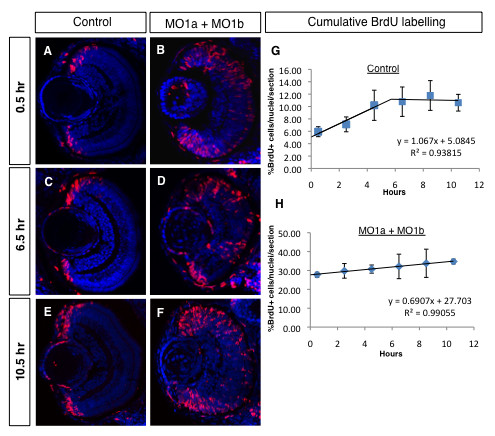
**Cumulative BrdU labelling reveals an increase in the morphant progenitor cell cycle in the 72 hpf retina**. (A-F) Immunolabelled coronal sections (dorsal to the top, lens to the left) from embryos repeatedly injected with BrdU (red) after different survival times (indicated on the left). Sections were counter-stained with DAPI (blue). (G, H) Regression analyses from cumulative labelling data. MO, morpholino; hpf, hours post-fertilization, hr, hours.

An analysis of cell proliferation in the retina at 72 hpf yielded similar results. PCNA expression in the double morphants was greatly expanded toward the central retina from the CMZ (Figure 7F), compared to the relatively few PCNA+ cells in the CMZ of control retinas (Figure 7E) and this was similarly confirmed with BrdU labelling (Figure 8, discussed below). Given that the retina is smaller in the morphants, an increase in PCNA+ and BrdU+ cells suggests that these cells might be delayed or stalled in the G1/S transition of the cell cycle. We examined the expression of phospho-histone H3 (pHH3), which labels cells in M-phase of the cell cycle, and found that there was approximately a two-fold increase in the number of pHH3+ cells [Control (n = 3): 15.3 ± 4.7 vs. MO1a + MO1b (n = 3): 28.0 ± 3.4; t = 3.7, p < 0.05] in transverse sections of the double morphant retinas primarily localized to the apical domain if the CMZ and central retina (Figure 7H) compared to controls (Figure 7G). Thus, cells are able to progress beyond S-phase of the cell cycle. We were also able to rule out that progenitor cells were undergoing endoreduplication of their DNA by using propidium iodide (PI) labelling and flow cytometry to quantify the average DNA content among a population of retinal cells from 72 hpf embryos injected with MO1a + MO1b and compared to cells from un-injected control embryos of equivalent age. We pooled dissected retinal tissue from 120 embryos in both groups. The vast majority of un-injected retinal cells at this stage (89.3%) were within G1 of the cell cycle, whereas only 5.7% were in S phase (Figure 7I). In contrast, 61.4% of the morpholino injected cells were in G1 phase of the cell cycle and 27.3% were in S phase (Figure 7J). The proportion of cells in G2-M of the cell cycle in either group was < 1% and this is due to the fact that the overall fraction of the cell population captured in these short phases of the cell cycle is rather low using this method. Nonetheless, there is a ~6.7 fold increase (from 0.07 to 0.47) in the proportion of cells in G2-M in the morphant retinas. Importantly, there was no evidence of polyploidy in either the control samples or the morphant samples. Therefore, these data indicate that retinal progenitor cells (and by inference midbrain cells) in morphant embryos are not stalled in any particular phase of the cell cycle and that they complete mitosis. Taken together, the smaller retinal size and increased proportion of cells that remain in cycle at 72 hpf suggests that progenitor cell cycle length is significantly increased.

To precisely quantify potential changes in cell cycle length in the *dmbx1 *double morphants at 72 hpf, we performed a BrdU cumulative labelling experiment (see Materials and Methods section for details) focusing on the retina. A 5 mM bolus of BrdU was given by intracerebroventricular (ICV) injection with survival times ranging from 0.5 hours to 10.5 hours at 2-hour intervals. For example, embryos in the 0.5 hour group would receive only a single BrdU injection and then processed for immunolabeling after 30 min. In contrast, embryos in the 10.5 hour group would have received a total of 6 separate injections (2 hours apart) and then processed for immunolabeling 30 min after the last injection. In preliminary experiments we determined that embryos receiving 6 repeated ICV injections remained viable and healthy (data not shown). The central assumption in this analysis is that an asynchronously dividing population of cells exhibits single population kinetics (i.e. all cells in the population have the same cell cycle time). Representative confocal images of BrdU labelled cells in control retinas at 0.5 hr, 6.5 hr, and 10.5 hr are shown in Figure 8A,C,E where proliferating cells are exclusively confined to the region of the CMZ. In contrast, in *dmbx1 *double morphant retinas, BrdU+ cells appear scattered through the peripheral and central retina (Figure 8B,D,F). Proliferating cells entering S-phase over time will incorporate BrdU and become labelled until they re-enter S-phase, at which point they can still incorporate BrdU, but they will not be marked as newly positive cells. In controls, the data during this interval (~0 - 5.5 hr) were fit to a linear regression model (R^2 ^= 0.93815), which allowed us to estimate when the maximal number of BrdU+ cells in the population were labelled (the first time point when the plateau is reached) (Figure 8G). Thus, by ~ 5.5 hours of cumulative BrdU labelling, all of the cells that are cycling in the population (the growth fraction) are labelled and further incorporation of BrdU at later time points does not increase this value. This allowed us to estimate the growth fraction in the controls to be ~11%. Using these values obtained from the plotted data (Figure 8G), we were able to estimate the progenitor cell cycle to be ~ 10.5 hours in the control retina (see Material and Methods for calculation).

The same analysis for *dmbx1 *double morphant retinas resulted in a significantly different cell cycle estimate. First, the fraction of cells incorporating BrdU over time continued to increase over the entire labelling interval (R^2 ^= 0.99055; Figure 8H). Therefore, were not able to accurately determine the growth fraction for the 72 hpf morphant retina, which would have required continuing the cumulative BrdU labelling well beyond 10 hours. However, we reasoned that the last time point assayed (10.5 hours) could be used as a minimum estimate for the time at which the growth fraction (i.e. ~35%) is reached (Figure 8H). Therefore, a minimal estimate for the cell cycle in these morphant progenitor cells is ~50.6 hours, which is approximately 5-fold longer than in the control retina. This increase in cell cycle length could account for the fact that the size of the retina at 72 hpf is significantly smaller since on average progenitor cells in the morphant retinas would not have completed one cell division between 48 hpf and 72 hpf. Our data indicate that a reduction in Dmbx1 proteins causes a significant increase in the cell cycle time of progenitor cells in the retina (and by inference the optic tectum) resulting is fewer differentiated cells.

### Rescue of the zebrafish morphant phenotype

In order to confirm the specificity of our knockdown phenotype, we co-injected either MO1a or MO1b morpholino with the corresponding zebrafish full-length mRNA (lacking the morpholino binding sequence). Microinjection of either *dmbx1a *mRNA or *dmbx1b *mRNA resulted in a significant dose-dependent dorsalization phenotype that was evident in embryos as early as tailbud stage and confirmed by 24 hpf (Additional file 6A; unpublished observations). We therefore, titrated the mRNA and evaluated the percentage of embryos co-injected with optimal amounts of mRNA and morpholino that demonstrated a rescued morphant phenotype. The highest concentration of either *dmbx1a *or *dmbx1b *mRNA (250 pg) caused mild to severe dorsalization in ~70 - 75% of the embryos injected and that ≥ 50% of these were in the severe category (Additional file 6B). Because some of these embryos showed signs of necrosis at 24 hpf, in particular in the tail region, we opted for a lower concentration of mRNA (150 pg for *dmbx1a *and 188 pg for *dmbx1b*, yielding similar phenotypic results) in order to test whether the paralog specific mRNA could rescue the morphant phenotype (see below). Using this lower concentration, ~ 50-70% fewer embryos were severely dorsalized (Additional file 6B).

**Figure 9:**
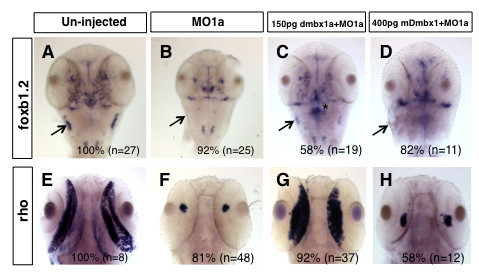
**Rescue of the midbrain and retinal phenotype in morphants is species-specific**. The expression domain of *foxb1.2 *in un-injected 48 hpf embryos (A) and *rho *in un-injected 72 hpf embryos (E) is significantly reduced in the MO1a injected embryos (B, F). Co-injection of zebrafish *dmbx1a *significantly rescues these phenotypes (C, G), whereas co-injection of mouse *Dmbx1 *mRNA does not (D, H). Arrow in A-D demarcates the region of the rostrolateral hindbrain (where the EG is located) at 48 hpf. For A-D, images represent dorsal views, anterior to the top. For E-H, images represent ventral views, anterior to the top. MO, morpholino; hpf, hours post-fertilization. Asterisk in (C) demonstrates ectopic *foxb1.2 *expression, which was commonly observed in embryos over-expressed with zebrafish *dmbx1a*, but not mouse *Dmbx1 *mRNA.

In a separate set of experiments we tested whether zebrafish *dmbx1a *mRNA could rescue the zebrafish MO1a morphant phenotype more by analyzing *foxb1.2 *and *rho *gene expression. The vast majority of embryos injected with MO1a resulted in a reduction of *foxb1.2 *expression in the midbrain and a loss of *foxb1.2 *expression in the rostrolateral hindbrain compared to the un-injected controls (Figure 9A,B). Furthermore, similarly treated embryos had dramatically reduced *rho *expression in the retina compared to un-injected controls (Figure 9E,F). By co-injecting zebrafish *dmbx1a *mRNA with MO1a, we were able to rescue the expression of *foxb1.2 *in the midbrain and rostrolateral hindbrain in ~50% of the injected embryos (when compared to the MO1a only group), as well as rescue the expression of *rho *in the retina by over 70% of the injected embryos (when compared to the MO1a only group) (Figure 9C,D). We also noticed that in embryos injected with zebrafish *dmbx1a *mRNA (especially without co-injection of MO1a) that there was a marked increase in *foxb1.2 *expression in the midbrain and rostromedial hindbrain (e.g. asterisk in Figure 9C; data not shown). Although we cannot completely rule-out subtle morpholino off-target effects, our data strongly suggest that the morphant phenotype is specifically due to the reduction in *dmbx1 *gene expression.

We also tested if the *dmbx1b *gene can rescue the *dmbx1a *morphant phenotype by examining *foxb1.2 *expression in the brain. As shown above, *foxb1.2 *is expressed in the dorsal midbrain and in the anterolateral hindbrain (in the region of the EG) and this pattern is similar to *dmbx1a*. In contrast, *dmbx1b *is not expressed in this anterolateral hindbrain domain, although it is expressed in the dorsal midbrain. Knockdown of *dmbx1a*, but not *dmbx1b*, results in the loss of *foxb1.2 *expression in the anterolateral hindbrain (Figure 4B,D,F). Therefore, we reasoned that if *dmbx1b *was capable of rescuing the *dmbx1a *morphant phenotype, then the expression of *foxb1.2 *in the anterolateral hindbrain would be restored. The results showed that while *dmbx1a *mRNA could rescue the midbrain and anterolateral hindbrain *foxb1.2 *expression in ~75% of the embryos co injected with MO1a (Additional file 7C), *dmbx1b *mRNA co-injected with MO1a was completely ineffective at restoring *foxb1.2 *expression in the anterolateral hindbrain, although midbrain expression was enhanced to control levels in ~50% of the embryos (Additional file 7D). We conclude that the functionality of the *dmbx1 *paralogs, in this experimental context, is not completely interchangeable.

Given the coding-sequence conservation between the mammalian *Dmbx1 *genes and the teleost *dmbx1 *genes, we also examined whether the full-length mouse *Dmbx1 *mRNA (lacking the morpholino binding site) could rescue the MO1a phenotype. The mouse *Dmbx1 *mRNA yielded a comparable dorsalization phenotype only after a 2-3 fold relative increase in mRNA was injected (Additional file 6C). However, the mouse *Dmbx1 *mRNA cannot rescue the *foxb1.2 *morphant expression in the midbrain and anterolateral hindbrain (Figure 9D), or the *rho *morphant expression in the retina (Figure 9H), as efficiently as the zebrafish mRNA. There were ~20% of the embryos co-injected with mouse mRNA and MO1a that had a slight increase in *rho *expression (compared to the MO1a only group), but this was substantially less than the control levels of expression (data not shown). We observed similar results when comparing the MO1b rescue with zebrafish *dmbx1b *compared to mouse *Dmbx1 *mRNA (data not shown).

It is possible that the mouse *Dmbx1 *mRNA is less stable in a zebrafish embryo accounting for the reduced potency of mouse *Dmbx1 *to induce a dorsalized phenotype. Unfortunately, currently available commercial antibodies for Dmbx1 do not recognize the zebrafish proteins (data not shown). Therefore, in order to address whether the mouse mRNA was less stable, we generated *c-myc *epitope-tagged mouse *Dmbx1 *and zebrafish *dmbx1a *constructs and monitored protein levels by immunohistochemistry using an anti-c-myc antibody at tailbud after injecting into 1-2 cell stage embryos. Compared to un-injected controls (Additional file 8A), embryos injected with either mouse *myc-Dmbx1 *(Additional file 8B) or zebrafish *myc-dmbx1a *(Additional file 8C) resulted in protein expression. Similar observations were made with injected 24 hpf embryos (data not shown). There was some variability in the overall intensity of staining between embryos regardless of the construct used, but in all cases injected embryos had discernable nuclear staining compared to un-injected controls (Additional file 8D-F). Thus, although subtle differences in mRNA stability may not be detectable in this assay, these data suggest that there is no overt difference in the mRNA stability of zebrafish and mouse mRNA that would account for the inability of the mouse *Dmbx1 *to rescue midbrain and retinal morphant gene expression phenotypes.

A limitation in our morphant rescue analyses relates to the dorsalization phenotype. The rescue of the brain and retinal morphant phenotypes with zebrafish *dmbx1 *mRNAs is consistent with the fact that there is a significant loss of function phenotype in these specific tissues. However, the rescue of the overall mRNA-induced dorsalization phenotype with co-injection of morpholino is more difficult to interpret. The onset of *dmbx1 *expression occurs as early as 9 hpf and the expression domain is strongest on the dorsal side close to the blastoderm margin [7,8]. It is possible that in addition to affecting the development of the midbrain territory, changes in the relative levels of *dmbx1 *expression at this early time might also indirectly affect the morphogenesis of tissues adjacent to the *dmbx1 *domain (e.g., convergence-extension defects) resulting in dorsalized embryos with high concentrations of mRNA. The co-injection of morpholino and zebrafish mRNA is presumably sufficient to mitigate these early morphogenetic defects. However, this hypothesis remains to be tested. In contrast, the co-injection of morpholino and mouse mRNA may not mitigate these early defects if the downstream effects of ectopic mouse mRNA are different. In other words, the dorsalization phenotypes from zebrafish and mouse mRNA injections might be caused by distinct molecular mechanisms. Further experiments are required to better understand the underlying mechanisms that might account for the dorsalization defects. Nonetheless, we also know that lowering the concentration of injected *dmbx1a *mRNA tends to significantly reduce the number of dorsalized embryos (Additional file 6), yet we are still able to observe a rescue of the *foxb1.2 *expression in the brain of MO1a morphants (preliminary data not shown). Thus, overall these data suggest that we can achieve a reliable rescue of the zebrafish MO1a and MO1b morphant phenotypes with corresponding zebrafish *dmbx1 *mRNAs.

### Molecular evolutionary analyses of vertebrate Dmbx1 genes

The morphant rescue results prompted us to examine whether there are any changes in the protein coding sequences among the vertebrate *Dmbx1 *genes that might correlate with the putative functional differences that we observed between the zebrafish and mouse genes. The results of a variety of phylogenetic analyses, including neighbour-joining, maximum likelihood and Bayesian methods, were largely congruent with current understanding of phylogenetic relationships among vertebrates [41-44]. These analyses strongly support a fish-specific *Dmbx1 *duplication event (Additional file 9), which we previously proposed [7] and may be associated with a postulated ancient genome duplication event early in the teleost lineage of fishes [45].

Determining the proportion of non-synonymous to synonymous (dN/dS) changes in the coding sequences of *Dmbx1 *genes allowed us to estimate the rate of amino acid evolution as well as make inferences about any changes in the selective constraints during the evolution of this gene family in vertebrates [46]. The results of our molecular evolutionary analyses of *Dmbx1 *genes suggest that although this family is generally quite conservative in its evolution, with an overall dN/dS of only 0.036, there was a dramatic change in selective constraint after the duplication event which gave rise to the *dmbx1a *and *dmbx1b *families in fish (Additional file 10). The elevation in dN/dS after this gene duplication is particularly marked in the lineage leading to *dmbx1a *(dN/dS = 46.88), suggestive of positive selection; whereas estimates along the *dmbx1b *lineage (dN/dS = 0.37), which are much lower, would be consistent with either weak positive selection or relaxed purifying selection. However, these results are based on analyses of a fairly small data set, which is particularly deficient in basal fish Dmbx1 genes; the inclusion of additional sequence data will improve the ability of these statistical methods to detect changes in the form and strength of selection across the Dmbx1 gene family. Additionally, *in vivo *assays of protein function, as we have done, are necessary to confirm functional divergence following the gene duplication event.

## Discussion

### The role of Dmbx1 in regulating brain and retinal neurogenesis

Our present results expand upon previous zebrafish *dmbx1a *knockdown experiments [8] to demonstrate that the *dmbx1 *paralogs have evolved both cooperative and divergent functions in brain neurogenesis. In the midbrain, particularly in the TeO, there is an independent, non-redundant requirement for both *dmbx1a *and *dmbx1b *functions, even though there is substantial overlap in progenitor cell expression of these genes. In contrast, divergent expression of the *dmbx1 *paralogs, particularly in the anterior hindbrain, correlate with divergent, paralog-specific (i.e. *dmbx1a*) function in neurogenesis. We reveal for the first time that the zebrafish *dmbx1 *genes have a fundamental role in regulating either the normal pace of progenitor cell cycle progression and/or the ability to exit the cell cycle and differentiate at the appropriate time. Further investigations will be required to ascertain the precise mechanism by which Dmbx1 proteins influence cell cycle timing.

A recent study demonstrated that *zic2a *and *zic5 *paralogous genes are required for TeO neurogenesis as early as 15 hpf, and that transcription of these genes is directly regulated by canonical Wnt signalling [47]. The earlier effect of *zic *gene expression on tectal proliferation suggests that *dmbx1 *function may be downstream of *zic*. One plausible model is that *zic *genes promote cell cycle entry from G0 in cells with a normal cell cycle, while *dmbx1 *genes promote cell cycle progression or exit. Given that these genes encode transcription factors, this role in cell cycle regulation could be indirect through the expression of cell cycle genes, such as *cyclind1 *[48] or *cdc16/26 *of the APC complex [49], but direct regulation of cell cycle proteins is also possible. Our preliminary yeast-2-hybrid analysis recovered Zic2b as a putative interacting partner with Dmbx1a, among other proteins with known functions in cell cycle regulation (L.W. and V.T., unpublished observations). Thus, it is possible that *dmbx1 *genes, *zic *genes and other cell cycle regulators physically interact in a protein complex to regulate transcription and/or other functions. Our data is consistent with the function of other vertebrate paired-like homeodomain transcription factors, such as *Chx10 *(*Vsx2*) and *Prox1*, which have been shown to play an important role in retinal progenitor cell cycle and differentiation [50]. Interestingly, a putative *Drosophila *ortholog *Pph13/Mu*, which is expressed in photoreceptor cells, has been shown to be required for differentiation and function, but not retinal progenitor cell specification [14]. One appealing model is that canonical cell cycle inhibitors interact with Dmbx1 and other factors to activate transcription of genes that promote cell differentiation and/or to repress genes that maintain a progenitor cell state.

Patterning and early morphogenesis of the midbrain, prior to neurogenesis, requires the establishment of organizing boundaries at the rostral extent (the diencephalic-midbrain boundary, DMB) and caudal extent (MHB) of the midbrain territory. *Otx2 *and *gbx2 *are two early expressing genes that pattern the neural plate rostrocaudally [51,52] and the interface of the two expression domains gives rise to the MHB, an organizer that activates a cascade of downstream transcriptional regulators, such as *pou2*, *pax2a*, *pax5/8*, and *eng2/3*, through Fgf8 signalling [53-55]. The DMB is established by the mutual repression of the forebrain marker *pax6 *and midbrain marker *eng2 *[56,57]. Consistent with previous loss of function data in both zebrafish [8] and mouse [9], neither *dmbx1a *nor *dmbx1b *is essential for MHB or DMB formation. Indeed, although the overall size of the midbrain territory is reduced, the expression of *dmbx1a*, *dmbx1b*, *otx2 *and *wnt1 *are normally regionalized in the double morphant embryos, indicating that Dmbx1 is not essential for brain patterning, but is instead an important regulator for subsequent neurogenesis.

Zebrafish *dmbx1a *and *dmbx1b *are both required for *foxb1.2 *expression in the midbrain and hindbrain. Although we have not determined whether this interaction is direct via transcriptional regulation of the *foxb1.2 *gene, this data suggests that the function of Dmbx1 may be mediated through *foxb1.2*. *Dmbx1 *has previously been shown to act as a transcriptional repressor [58], therefore it is possible that the mechanism for regulating *foxb1.2 *expression may involve an indirect de-repression mechanism. Interestingly, the relationship between the *Dmbx1 *and *Foxb1 *orthologs in mouse is different. In mouse, a null mutation in the orthologous *Foxb1 *gene affects midbrain neurogenesis, particularly within the inferior colliculus, which is most notable in the postnatal period with little or no effect on the development of the superior colliculus (the homolog of the fish TeO) [59,60]. In contrast, there is no discernable embryonic or postnatal defect in the *Dmbx1 *null midbrain and there is no change in *Foxb1 *gene expression in the *Dmbx1 *null embryos [9]. This is strikingly different from our observations that single knockdowns of either *dmbx1a *or *dmbx1b *causes a significant growth and differentiation defect in the midbrain, which is strongly correlated with a significant reduction in *foxb1.2 *gene expression. These observations lead us to speculate that there is post-duplication evolutionary divergence of function in teleosts in which *dmbx1 *genes have a more prominent role in regulating neurogenesis compared to other vertebrate classes that harbour a single *Dmbx1 *gene, possibly through the novel regulation of *foxb1 *genes. However, we cannot yet rule out that other components regulating neurogenesis in mice may have been functionally compensating in the *Dmbx1*knockout, and hence masking a phenotype that would otherwise resemble the zebrafish *dmbx1 *knockdown phenotype. Post-duplication functional divergence has also been proposed for the zebrafish *midkine *genes [61], and so it would be interesting to compare their functional evolution with that of the *dmbx1 *genes to determine if similar underlying mechanisms have evolved.

The Dmbx1 loss of function phenotype in the retina (extended cell cycle length and reduced differentiation) resembles the *disarrayed *[62] and *caf-1b *[63] mutants, where both cell cycle progression and/or exit, and differentiation, are significantly delayed during retinal development. However, in both of these mutants there is a substantial increase in apoptosis between 42 hpf and 65 hpf (for *disarrayed*), or 48 hpf and 72 hpf (for *caf-1b*). Interestingly, rescue of apoptosis with a *p53 *morpholino did not rescue the differentiation defect in the *caf-1b *mutants [63], consistent with the possibility that a delayed cell cycle defect (independent from the cell survival defect) prevents differentiation, similar to what we observe in *dmbx1 *morphants. In contrast, we did not observe persistent cell death in the brain or retina after 24 hpf in the *dmbx1 *morphants. Nonetheless, it would be interesting to further characterize the network of genes that regulate the transition from a proliferating progenitor cell to a post-mitotic progenitor, for which *disarrayed*, *caf-1b *and *dmbx1 *genes may be required.

Dmbx1 appears to have an essential role in the differentiation of the rod and cone photoreceptor lineages. Interestingly, double morphant embryos had enhanced expression of the basic helix-loop-helix transcription factor gene *neurod*. It has been recently shown that *neurod *expression marks early progenitor cells within the INL that predominately give rise to rod (and cone) precursor cells that express *crx *and traverse the ONL to eventually differentiate into mature photoreceptors [35]. Both Dmbx1 paralogs are expressed in the INL during photoreceptor lineage development, like Neurod1 and Crx [35,64]. However, *dmbx1 *expression does not appear to overlap with *neurod1 *and *crx *in the ONL. Thus, we speculate that Dmbx1 functions in parallel (or upstream) of *neurod *and *crx *in regulating photoreceptor progenitor cell cycle exit and/or the onset of photoreceptor differentiation.

### The evolution of vertebrate Dmbx1 genes

Studies in zebrafish are increasingly playing an important role in deciphering the functional consequences of gene duplication. In this regard, significant insight into the fundamental changes in brain development that are due to the retention of duplicate genes in zebrafish comes from studies of the *Dlx *gene family [65,66], *Hoxb *genes [67,68], the *Zic *gene family [47,69] and *Pax6 *[28]. In most cases, redundancy or subfunctionalization is reported to play an important role in the retention of duplicate genes. However, these mechanisms may not result in substantial genomic novelty to account for the developmental specializations during vertebrate neural evolution [70]. Additional mechanisms for duplicate gene retention, such as neofunctionalization [63] or function shuffling [26] may have evolved, and may or may not be operating on different gene duplicates within a single genome [30]. Therefore, it seems reasonable to assume that the function of individual genes of a duplicate pair will have subtle and complex roles in brain development.

The *Dmbx1 *gene is also conserved in the *Amphioxus *and ascidian genomes, but is either not expressed in the nervous system during development (as in *Amphioxus*) or is expressed in a domain that is posterior to the developing sensory vesicle and neck (*pax2/5/8a-*expressing domain) region of the neural tube starting at approximately tailbud stage in ascidians [71-73]. These observations indicate that the *Dmbx1 *neural expression domain in invertebrate chordates is likely to be homologous to part of the vertebrate hindbrain domain, consistent with the notion that a true midbrain region is absent in these species [72]. Thus, *Dmbx1 *expression in a region that is anterior to the midbrain-hindbrain boundary (MHB) demarcates a distinct midbrain territory hypothesized to be a derived anatomical trait that evolved specifically in vertebrates.

Our findings suggest zebrafish Dmbx1 is required for proper cell cycle progression or exit and cell differentiation of progenitor cells in the brain and retina. Furthermore, several observations, including: (1) the differences in gene expression pattern, (2) loss of function phenotypes, (3) lack of complete interchangeable function in rescue experiments, and (4) the changes in selective constraints in protein sequence evolution, suggest that the post-duplication zebrafish *dmbx1 *genes may have evolved a diverged function during neural development.

Since our present study primarily focused at the level of cell/tissue/development for our analyses, further studies are required to resolve whether changes at the amino acid level have direct consequences for protein function. A recent analysis of the statistical methods used to determine positive selection in protein coding sequences questions the reliability of some models, such as the branch-site model not used in our analyses, in their ability to predict positively selected sites and suggests that experimental confirmation would be necessary in such analyses [74]. Our evidence of positive selection in the teleost *dmbx1 *coding sequences is consistent with the differences in functionality between zebrafish and mouse *Dmbx1 *that we observed in our morphant rescue experiments. Does this evidence support the possibility for neofunctionalization [75]? It is tempting, but premature to answer this question in the affirmative. In order to substantiate such a model we need to understand the function of the Dmbx1 proteins in various vertebrate species in much more detail in order to deduce which evolutionary mechanism could plausibly explain the retention of the Dmbx1 paralogs in the teleost genome. Nonetheless, our data provide a reasonable basis to further investigate this problem experimentally.

## Conclusion

There are four main observations from our study of the functional role of *dmbx1 *duplicate genes in zebrafish development. First, both *dmbx1a *and *dmbx1b *are independently required, and hence cooperate in regulating neurogenesis in the midbrain. Second, *dmbx1a *has a predominant role in regulating neurogenesis in the retina and anterior hindbrain, and is therefore partially functionally diverged from *dmbx1b*. Third, the cellular mechanism of zebrafish *dmbx1 *function is to control cell cycle exit and/or differentiation in progenitor cells. Finally, we provide evidence for post-duplication positive selection in teleost *dmbx1 *genes that correlate with differences in over expression/rescue phenotypes between the zebrafish and mouse *Dmbx1 *genes. Therefore, zebrafish *dmbx1 *duplicate genes may be functionally diverged and appear to have an important role in regulating the transition from a proliferating progenitor cell to a post-mitotic differentiated neural cell.

## Methods

### Zebrafish husbandry

Adult zebrafish (Danio rerio) used in this study were maintained at 28°C on a 14-hour light/10-hour dark cycle and housed in an automated re-circulating system (Aquaneering). Animals were treated in accordance with the regulations on animal experimentation established by the Canadian Council on Animal Care. The experimental procedures were approved by the University of Toronto Animal Care Committee. Embryos were staged as described in Kimmel et al. [76] and reared according to standard procedures [77]. The wildtype strain used was AB (Zebrafish International Resource Center) and the Tg(isl2b:GFP)^zc7 ^transgenic strain was a kind gift from Dr. Chi-Bin Chien.

### GFP fusion proteins

Primers were designed to flank part of the 5'-UTR and N-terminal domains of *dmbx1a *and *dmbx1b *that are complementary to the morpholino sequences. The previously described *dmbx1a*-MO (MO1a) was complementary to the sequences surrounding the ATG start codon [8] and the corresponding fusion protein was generated by cloning 70 bp of the 5'-UTR and the first 16 amino acids of Dmbx1a in frame with the EGFP gene (Dmbx1a-FP primers: F:5'-CGAGCTAGAAGCAAGAAAATATCA-3' and R:5'-GAGTTCATGGCGTGGAGAGAGTA-3'. The *dmbx1b*-MO (MO1b) targeted the 5'-UTR sequences just upstream of the start codon. The fusion protein consisted of the 99 bp of the 5'-UTR plus amino acids 1-16 of Dmbx1b, followed by the EGFP gene sequences (Dmbx1b-FP primers: F:5'-TGGGAAAAATCACTCGTGTTC-3' and R: 5'-GAGTTCATGGCGTGCAAA-3'). The PCR fragments of dmbx1a-FP and dmbx1b-FP were cloned upstream and in frame with EGFP in pCS2^+^. Plasmids were linearlized with BstX1 and in vitro transcribed with the SP6 messenger kit (Ambion). For each fusion construct, 500 pg of mRNA was injected at the 1-cell stage embryo in the presence or absence of morpholinos.

### BrdU labelling

To label cells that were in S-phase, we injected 5 mM of 5'-bromo-2'-deoxyuridine (BrdU) into the tectal brain ventricle of 72 hpf embryos and fixed the animals (n = 10) with 4% paraformaldehyde half and hour later. For cumulative cell cycle analysis using BrdU incorporation, embryos (n = 12) were injected with 5 mM BrdU every two hours up to 10 hours followed by 4% paraformaldehyde fixation 30 minutes post-BrdU injection. Cyrosectioning procedures were performed as mentioned above. For BrdU-immunostaining, slides were treated with 20U/mL DNase I at room temperature for 30 minutes followed by extensive washes with PBS+1% DMSO+0.1% Tween-20 (PBDT). Sections were blocked for two hours and incubated in rat anti-BrdU (1:100, Cedarlane) primary antibody overnight at 4°C, which was then detected with Cy3 secondary antibody (1:500, Jackson ImmunoResearch Laboratories, Inc.). Images were obtained from mounted slides using Leica TCS SP5 II Confocal Microscope and analyzed with Leica LAS AF software. For the cumulative BrdU assay, we counted the number of BrdU positive cells per section (averaged over at least 3 separate retinas) and we used cell density (number of DAPI positive nuclei/area of section) to estimate the number of total nuclei from each section and calculated the labelling index (BrdU positive cells/total nuclei) at all six time points. Cell cycle kinetics in control and morphant embryos was determined as described in [78,79] assuming this was a single population model. Briefly, hours of BrdU injection (T) was plotted against the labelling index (LI). Growth fraction (maximum LI on the y-axis, LI_m_) can be determined from where the curve begins to plateau. The time when the maximum amount of BrdU positive cells was labelled is equal to total cell cycle time (Tc) minus S-phase time (T_s_). By extrapolating the curve back to time = 0, we can also find out the labelling index at T_s _(LI_0_). With this information, the total cell cycle time can be estimated using the equation LI_0_/LI_m _= T_s_/T_c_.

### Whole-mount in situ hybridization

Embryos treated with 0.003% of 1-phenyl-2-thiourea (Sigma) were fixed in 4% paraformaldehyde and processed as previously described [7]. The following antisense RNA probes were used: *eng2b *[80], *fgf8 *[81], *foxb1.2 *[82], *egr2b (krox20) *[83], *islet1 *[84], *pax2a *[85], *otx2 *[86] (kind gifts from Dr. Ashley Bruce); *dmbx1a*, *dmbx1b *and *wnt1 *(cloned from cDNA), and *erm, pea3 *[87] (kind gift from Dr. Herbert Steinbeisser); *neurod*, *opn1sw2*, *pax6a*, *rho*, and *vsx2 *(Open Biosystems). Embryos were cleared in glycerol before images were captured with a Leica MZ16F dissecting microscope (whole mounted samples) or a Leica DM4500B compound microscope (flat-mounted samples) with a QIMAGING digital camera and OpenLab software.

### Anitsense morpholino and RNA injection

Antisense morpholinos (MOs) were obtained from Gene Tools, Inc. Dmbx1a-MO was complementary to the sequences that flanked the ATG start cordon as previously described [8], whereas dmbx1b-MO was targeted to sequences upstream of the start cordon in the 5' UTR. The sequences of the MOs are as follows, *dmbx1a *MO (MO1a): 5'-ACTCCGTAGTGCTGCATGATTCACA-3' and *dmbx1b *MO (MO1b): 5'-TCGAGCTTCTCTCTGGGAAGTTTTG-3'. A 5-mismatched nucleotides MO was also synthesized for both *dmbx1a *(mMO1a): 5'-ACTgCGTAcTGCTcCATcATTgACA-3' and *dmbx1b *(mMO1b): 5'-TCcAGCTTgTCTgTGcGAAcTTTTG-3' as controls. Unless otherwise noted, embryos were injected with 10 ng of a single MO, or 5 ng each of the combined MOs into the yolk at 1- to 2-cell stages.

### Ectopic gene expression

Dmbx1aCDS primers (F:5'-ATGCAGCACTACGGAGTGAA-3' and R:5'-TCAGTTGGGCAGTGTGTCC-3') and Dmbx1bCDS primers (F:5'-ATGCAGCACTACGGGGTGA-3' and R: 5'-TTAGTTTGGTAGCGTGTCCAGG-3') amplified the full coding sequences of dmbx1a and dmbx1b, respectively, but which lack the 5'-UTR to avoid binding of the corresponding morpholinos. Both PCR fragments were cloned into pCS2^+ ^and linearized with SacII for in vitro RNA transcriptions using the mMESSAGE mMACHINE SP6 kit (Ambion). Mouse *Dmbx1 *mRNA was synthesized from pCMV6-Kan/Neo plasmid containing the cloned full length mouse Dmbx1 cDNA (OriGene), but lacked 5'-UTR sequences corresponding to morpholino binding sites. The template was linearized with SacII and transcribed using the mMESSAGE mMACHINE T7 kit (Ambion). N-terminal Myc-tagged mouse Dmbx1 was subcloned using EcoRI and XbaI from pCMB6Kan/Neo-Dmbx1 into the pCS2^+^-MT plasmid, whereas zebrafish N-terminal Myc-dmbx1a was generated by cloning klenow-treated BamHI+XhoI pCS2^+^-dmbx1aCDS into CIAP-treated (Invitrogen) XbaI digested pCS2^+^-MT plasmid. RNA for Myc-Dmbx1 and Myc-dmbx1a were synthesized by linearlizing both plasmids with NotI, and then transcribed with the mMESSAGE mMACHINE SP6 kit (Ambion). RNA or RNA+MO were injected into the yolk of 1-cell stage embryos at the concentrations indicated.

### Histology

Embryos were fixed in 4% paraformaldehyde and rinsed in phosphate-buffered saline solution after. Embryos were first dehydrated using increasing concentrations of ethanol, followed by embedding with increasing concentrations of Spurr's resin in ethanol. Embryos were then left to polymerization at 65°C in 100% Spurr's resin. Semithin coronal sections (approximately 1 μm thick) were cut with a glass knife using an ultramicrotome and dried onto glass slides. This procedure was followed by counterstaining with toluidine blue to visualize morphology. Whole-mount in situ hybridization embryos in 100% glycerol were washed with PBT and followed by the same embedding and sectioning steps as above. Sections were 1.5 micrometers thick without counterstaining to maximize visualization. To measure the area of the retina and optic tecta, five plastic sections with similar focal plane were chosen to represent each embryo, and images were taken on a Leica DM4500B compound microscope with a QIMAGING digital camera and OpenLab software. The areas of interest were outlined and measured using the program ImageJ (http://rsb.info.nih.gov/ij/) [88]. Results represent the average obtained from at least 5 embryos from each group. Statistical analyses between injected and un-injected groups were performed using student's t-test. Differences were regarded as significant for p < 0.05.

### Immunohistochemistry

Two-three dpf embryos from each group (n = 10) were fixed with 4% paraformaldehyde overnight at 4°C and washed in sucrose series (from 5% to 30% sucrose in PBS) for cryoprotection. For PCNA labelling, embryos were fixed in 37% formaldehyde:95% ethanol (1:9 ratio) solution. Samples were left in 30% sucrose:OCT (2:1 ratio) at -20°C before cutting into 10-15 μm sections with a cryostat. Sections were re-hydrated with 1×PBS and blocked for 2 hours in 0.2% Triton X-100 + 2% goat serum in PBS at room temperature. Primary antibody in block solution was applied on sections overnight at 4°C. Slides were washed with PBS + 0.1% Tween-20 and incubated with secondary antibody (1:100 Cy2 or 1:500 Cy3, Jackson ImmunoResearch Laboratories, Inc.) for 2 hours at 4°C. Nuclei were counterstained with DAPI before mounting the slides. *Tg(isl2b:GFP) *embryos were used to examine retinal ganglion cells and no staining was performed after cryosectioning. Phospho-histone H3 antibody was conjugated to Alexa Fluor^® ^488 so no secondary antibody was required. The following primary antibodies were used: mouse anti-PCNA (1:100, ZYMED Laboratories), rabbit anti-Phospho-histone H3 (Ser10) (1:100, Cell Signaling), rabbit anti-Pax 6 (1:100, Covance), rabbit anti-PKC (1:100, Santa Cruz Biotechnology, Inc.), mouse anit-GS (1:500, Chemicon), mouse anti-Zpr1 (1:200, ZIRC), and mouse anti-Zpr3 (1:200, ZIRC). Images were taken from Leica TCS SP5 II Confocal Microscope and analyzed with Leica LAS AF software. For phospho-histone H3 positive cell count, we obtained three sections from different embryos of each group and took the average number for comparison.

### Whole-mount antibody staining

Ten embryos were fixed in 4% paraformaldehyde at 4°C overnight and washed with PBS. Samples were incubated with block solution (PBS+1%BSA+1%DMSO+0.8% TritonX-100) for 1 hour at room temperature and overnight in anti-c-myc (9E10; Developmental Studies Hybridoma Bank) antibody (1:20) at 4°C. Embryos were then washed with PBS+Triton X-100 and incubated in goat anti-mouse HRP antibody (1:500; Jackson ImmunoResearch Laboratories, Inc.) overnight. Before DAB staining, samples were washed with PBS+Trition X-100 followed by PBS only. Peroxidase activity was detected with DAB and 3% hydrogen peroxide in the dark. Images of the stained embryos were taken with a Leica MZ16F dissecting microscope or a Leica DM4500B compound microscope.

### Cell death analyses

Embryos from 24 - 72 hpf (n = 10) were bathed in embryo media that contained 5 μg/mL acridine orange (AO; Sigma) for 15 min at room temperature, and immediately followed by 4×10 min washes using regular media. AO-positive cells were imaged using a Leica DM4500B compound microscope with a QIMAGING digital camera and OpenLab software. For TUNEL labelling, embryos at 72 hpf (n = 10) were fixed in 4% paraformaldehyde at 4°C overnight followed by cryoprotection with sucrose and cryosectioning (same as immunohistochemistry). Sections were rehydrated and TUNEL assay was carried out according to manufacturer's instruction (Apo-Direct TUNEL Assay Kit, Millipore) and images were captured using Leica TCS SP5 II Confocal Microscope and analyzed with Leica LAS AF software.

### Retinotectal projections

*Tg(isl2b:GFP) *larvae treated with 0.003% of 1-phenyl-2-thiourea (PTU) at 4dpf were immobilized on long cover slip and sandwiched with another smaller elevated cover slip in order to observe their retinotectal projections dorsally. Images were collected using Zeiss LSM 510 inverted confocal microscope.

### Flow cytometry

Retinas from 60-80 embryos (~2 × 10^6 ^cells/mL) were dissected out from sample and control groups and left in Hank's Buffered Salt Solution (HBSS) with trypsin for 30 minutes. Cells were centrifuged, supernatant was discarded, and pellet was re-suspended in 50 μL of HBSS with 2% FBS. We next added 1 mL of ice-cold 80% ethanol and kept samples in -20°C for at least 30 minutes. Cells were collected by centrifugation and washed twice with HBSS + 2% FBS. Removed supernatant and added 500 μL of 0.1 mg/mL propidium iodide (Sigma) in HBSS with 0.6% NP-40, together with 500 μL of 2 mg/mL RNaseA and incubated in the dark for 30 minutes. Samples were filtered through 85 μm Nitex mesh filter and analyzed with BD FACSAria™ cell sorter. Flow cytometry data were analyzed using FlowJo software.

### Phylogenetic analyses

An alignment of vertebrate Dmbx1 genes was assembled using BLAST searches of Ensembl (v.52) and NCBI (including both annotated genes and whole-genome shotgun reads). Amino acid-translated sequences were aligned using ProbCons [89], and trimmed using Gblocks (v0.91b; [90]) following the 'relaxed' trim procedure of [91]. This data set was subjected to a variety of phylogenetic analyses, including maximum likelihood, Bayesian inference, and neighbour-joining distance methods [92-94]. Maximum likelihood phylogenetic methods were implemented in the program PHYML v2.2.4 [93,95]. A WAG substitution model was assumed [96], including additional parameters for among-site rate heterogeneity (+G; [97]) and invariant sites (+I; [98]). A neighbour-joining tree [92]) was constructed in the MEGA4 program, with a Poisson distance correction [99]. For both the likelihood and neighbour-joining analyses, bootstrap analysis was used to assess the degree of confidence in nodes of the phylogeny [100]. 100 replicates were performed for the likelihood analysis, and 1000 replicates for the neighbour-joining analysis. Finally, Bayesian inference was performed in MrBayes 3.1.2 [94]. As with the likelihood methods, a WAG+I+G substitution model was assumed. For all parameters in the Bayesian analysis, uniform priors were used, except for branch lengths, for which exponential priors were implemented. Two independent analyses were performed, each composed of 4 Markov chains with default heating values. Markov chains were run until the average standard deviation of split frequencies, a measure of stationarity, dropped below 0.01, sampling trees (and parameters) every 1000 generations. Convergence was assessed using a number of methods. A convergence diagnostic for branch length posterior probabilities, the potential scale reduction factor (PSRF), roughly approached 1 as the runs converged [101]. Convergence to stationarity was also assessed by plotting log-likelihood scores and other parameter values in the program Tracer 1.4.1 to ensure that there were no trends in the data post burn-in [102]. Finally, adequacy of mixing was assessed by examining acceptance rates for parameters in MrBayes, and by calculating in Tracer effective sample sizes (ESS), the number of independent samples from the marginal posterior distribution for each parameter; higher values being indicative of better sampling from the posterior distribution. These values were all well above 100. By these measures convergence was achieved within the first 25% of trees sampled, which were discarded as burn-in, and remaining trees were taken as representative of the posterior probability distribution.

### Molecular evolutionary analyses

Maximum likelihood phylogenetic methods were used to estimate the ratio of non-synonymous to synonymous rates (dN/dS) along lineages [46,103] in a pruned *Dmbx1 *phylogeny consisting of a subset of the sequences used for the phylogenetic analysis. This phylogeny was not only consistent with our analyses of the larger *Dmbx1 *data set, it is also congruent with current understanding of relationships among vertebrate taxa, assuming a teleost-specific genome duplication event [45]. dN/dS ratios can be used to estimate the form and strength of selection operating in the *Dmbx1 *gene family. Assuming no selection pressure, coding sequences will evolve neutrally, and nonsynonymous and synonymous substitutions should accumulate at equal rates, resulting in a dN/dS value equal to one [104,105]. Positive (or diversifying) selection is indicated by dN/dS values greater than one, while negative (or purifying) selection is indicated by dN/dS values near zero. Codon models that allow for variation in dN/dS along branches were implemented in the PAML package (v4.2a; [106]). Likelihood ratio tests were used to determine which among nested models provided a statistically significantly better fit to the data at hand [97,107].

## Abbreviations

PBS: phosphate-buffered saline; PBT: PBS with Triton-X100; HCl: hydrochloric acid; dNTP: deoxy-nucleotide-triphosphates, where the nucleotide is an equal mixture of adenine, guanine, cytosine, and thymine; FBS: fetal bovine serum.

## Authors' contributions

LW contributed to the design of the experiments, performed all of the molecular experiments, immunolabeling, and microinjections and participated in the data analysis and drafting the manuscript. CK performed the semi-thin plastic sectioning. CJW and BSWC carried out the phylogenetic and molecular evolution analyses and analyzed the data. VT conceived of the study, and participated in its design, coordination and data analysis, drafted and edited the manuscript. All authors read and approved the final manuscript.

## Supplementary Material

Additional file 1 **Altered midbrain gene expression in morphant embryos**. Lateral (A-F, J-L) and dorsal (G-I, M-O) views anterior to the left, or anterior views (P-R) dorsal to the top of embryos either un-injected (A, D, G, J, M, P) or injected with MO1a + MO1b (B, E, H, K, N, Q) or control mMO1a + mMO1b (C, F, I, L, O, R). All embryos are at 48 hpf, except for J-L, which are at 24 hpf. Arrowhead in B and E indicates the reduced expression in the dorsal midbrain and arrow in E indicates reduced expression in the hindbrain. Arrowhead in G, H and M, N demarcates the position of the MHB. Arrowhead in P, Q indicates the optic stalk region. MO, morpholino; mMO, mismatch morpholino; MHB, midbrain-hindbrain boundary; hpf, hours post-fertilization. Click here for file

Additional file 2 **Midbrain-hindbrain boundary is unaffected in *dmbx1 *morphants**. Lateral view (anterior to the left) of embryos at 24 hpf (A-F, M-O) or 48 hpf (G-L). Analysis of genes normally expressed in the midbrain-hindbrain boundary region in un-injected (A, D, G, J, M), MO1a + MO1b injected (B, E, H, K, N), and mMO1a + mMO1b injected (C, F, I, L, O) embryos. Arrowheads demarcate the position of the midbrain-hindbrain boundary. MO, morpholino; mMO, mismatch morpholino; hpf, hours post-fertilization. Click here for file

Additional file 3 **Dose-dependent changes in *foxb1.2 *and *rhodopsin *gene expression in *dmbx1 *morphants**. Analysis of gene expression at 72 hpf in un-injected, MO1a injected, MO1b injected or MO1a + MO1b injected embryos using the MO concentrations listed. For *foxb1.2 *expression, embryos are shown in lateral view with anterior to the left. For *rhodopsin *expression, embryos are shown in ventral view, anterior to the left. Control embryos injected with mismatch MOs at similar concentrations showed no change in expression and are not shown. MO, morpholino; hpf, hours post-fertilization Click here for file

Additional file 4 **Distinct patterns of rhodopsin expression and retinotectal projections in *dmbx1a *and *dmbx1b *morphant embryos**. Dorsal view anterior to the top of un-injected (A, B), MO1a injected (C, D), MO1b injected (E, F), and MO1a + MO1b injected (G, H) embryos demonstrating expression of *rhodopsin *(*rho*) (A, C, E, G) or the retinotectal projection pattern (green fluorescence) as defined in *isl2b:GFP *transgenic embryos (B, D, F, H). The mismatch control injected morpholinos resembled the un-injected controls and are not shown. In B, D, F, H, white arrow demarcates the terminal field of the retinal ganglion cells in the optic tectum and the white arrowhead demarcates axonal fibers of the optic nerve. MO, morpholino. Asterisk demarcates the region of trigeminal ganglion. Click here for file

Additional file 5 **Cell death does not persist in *dmbx1 *morphant embryos**. Live embryos at 24 hpf (A, B, lateral view anterior to the left), 48 hpf (C, D, dorsal view anterior to the left) and 72 hpf (E, F, dorsal view anterior to the left; K, L, close up of retina lateral view dorsal to the top) were examined for the presence of apoptotic cells using AO. Arrows point to AO+ cells in the midbrain (bright spots). TUNEL+ cells (green) or Caspase3+ cells (red) on cryosectioned tissue of 72 hpf midbrains (G-J) and retina (M-P, arrow pointing to labelled cell) counter-stained with DAPI (blue). (MO, morpholino; mMO, mismatch morpholino; hpf, hours post-fertilization; AO, acridine orange. Click here for file

Additional file 6 **Zebrafish, but not mouse, *Dmbx1 *mRNA counteracts the zebrafish knockdown phenotype**. (A) Representative images of single embryos, lateral view anterior to the left from the different groups analyzed for a dorsalization phenotype. Embryos were injected with the mRNA concentrations shown, co-injected with 10 ng of either MO1a or MO1b as indicated, and scored for a dorsalized phenotype at 24 hpf. Tabulated results for all groups from 2-5 separate experiments using (B) zebrafish mRNA, or (C) mouse mRNA. MO, morpholino; hpf, hours post-fertilization. Click here for file

Additional file 7 **Partial rescue of *dmbx1a *morphant with *dmbx1b *mRNA**. (A) Un-injected and (B) MO1a injected embryos showing *foxb1.2 *expression at 48 hpf. Note, the data in these two panels are identical to panels (A) and (B) shown in Figure 9; they were duplicated here for ease of reference. Representative images of embryos co-injected with MO1a and either *dmbx1a *mRNA (C) or *dmbx1b *mRNA (D). Dorsal view of embryos, anterior to the top. Black arrows in all panels point to the anterolateral hindbrain region where *foxb1.2* is normally expressed. White arrows in all panels point to the dorsal midbrain. MO, morpholino. Click here for file

Additional file 8 **Monitoring mouse Myc-Dmbx1 and zebrafish Myc-dmbx1a levels in vivo**. Representative images of an (A) un-injected, (B) mouse *myc-Dmbx1 *mRNA injected (400 pg), and (C) zebrafish *myc-dmbx1a *mRNA injected (150 pg) tailbud stage embryo (lateral view) processed for whole-mount immunolabeling using an anti-Myc antibody (n = 10 per group). Panels D-F are representative high magnification images of embryos in each of the three treatment groups; black arrows indicate nuclei that are DAB+ in the injected groups, but lack DAB staining in the un-injected group. Click here for file

Additional file 9 **Phylogenetic analysis of vertebrate *Dmbx1 *genes**. Phylogram showing the neighbour-joining tree topology, with support values indicated for each node in the tree, for the three different phylogenetic methods of analysis employed. Above the nodes are neighbour-joining (1000 replicates), followed by likelihood bootstrap percentages (100 replicates); below the nodes are the Bayesian posterior probabilities. Bootstrap percentages below 50% are indicated by an asterisk. Click here for file

Additional file 10 **Molecular evolutionary analysis of vertebrate *Dmbx1 *genes**. Proportions of non-synonymous to synonymous rates (dN/dS) along lineages in the Dmbx1 phylogeny were estimated using codon-based maximum likelihood phylogenetic methods. Results of a branch model in which the post-duplication branches (PDBs) leading to the *dmbx1a *and *dmbx1b *clades were each allowed to have independently estimated dN/dS values are shown. Both PDBs display elevated dN/dS estimates compared to the background estimate applied to the remainder of the phylogeny. This model fit the data significantly better than a model with a single dN/dS parameter (M0 model; p < 0.01, d.f. = 2). Increases in dN/dS along each of the PDBs were also confirmed through simpler branch models in which only a single PDB, either for *dmbx1a *or *dmbx1b*, received separately estimated dN/dS values; in both cases the increases in dN/dS was statistically significant (M0; p < 0.05, d.f. = 1). Branch lengths shown in this figure were estimated under the 2 PDB branch model under which the dN/dS estimates were derived, and are proportional to the number of substitutions per codon. Click here for file
