# Supplementary figures and images for: Duplicate dmbx1 genes regulate progenitor cell cycle and differentiation during zebrafish midbrain and retinal development
Source: BMC Dev Biol. 2010 Sep 22;10:100. doi: 10.1186/1471-213X-10-100 (PMC2954992; doi:10.1186/1471-213X-10-100)

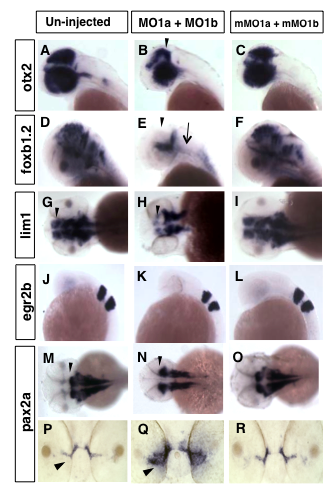

Supplement: Additional file 1 — Altered midbrain gene expression in morphant embryos. Lateral (A-F, J-L) and dorsal (G-I, M-O) views anterior to the left, or anterior views (P-R) dorsal to the top of embryos either un-injected (A, D, G, J, M, P) or injected with MO1a + MO1b (B, E, H, K, N, Q) or control mMO1a + mMO1b (C, F, I, L, O, R). All embryos are at 48 hpf, except for J-L, which are at 24 hpf. Arrowhead in B and E indicates the reduced expression in the dorsal midbrain and arrow in E indicates reduced expression in the hindbrain. Arrowhead in G, H and M, N demarcates the position of the MHB. Arrowhead in P, Q indicates the optic stalk region. MO, morpholino; mMO, mismatch morpholino; MHB, midbrain-hindbrain boundary; hpf, hours post-fertilization. [file 1471-213X-10-100-S1.TIFF]

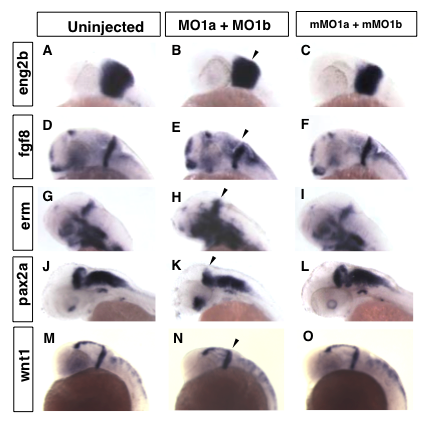

Supplement: Additional file 2 — Midbrain-hindbrain boundary is unaffected in dmbx1 morphants. Lateral view (anterior to the left) of embryos at 24 hpf (A-F, M-O) or 48 hpf (G-L). Analysis of genes normally expressed in the midbrain-hindbrain boundary region in un-injected (A, D, G, J, M), MO1a + MO1b injected (B, E, H, K, N), and mMO1a + mMO1b injected (C, F, I, L, O) embryos. Arrowheads demarcate the position of the midbrain-hindbrain boundary. MO, morpholino; mMO, mismatch morpholino; hpf, hours post-fertilization. [file 1471-213X-10-100-S2.TIFF]

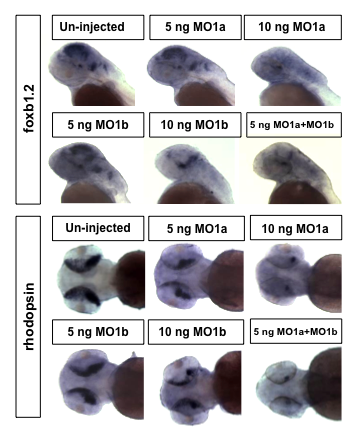

Supplement: Additional file 3 — Dose-dependent changes in foxb1.2 and rhodopsin gene expression in dmbx1 morphants. Analysis of gene expression at 72 hpf in un-injected, MO1a injected, MO1b injected or MO1a + MO1b injected embryos using the MO concentrations listed. For foxb1.2 expression, embryos are shown in lateral view with anterior to the left. For rhodopsin expression, embryos are shown in ventral view, anterior to the left. Control embryos injected with mismatch MOs at similar concentrations showed no change in expression and are not shown. MO, morpholino; hpf, hours post-fertilization [file 1471-213X-10-100-S3.TIFF]

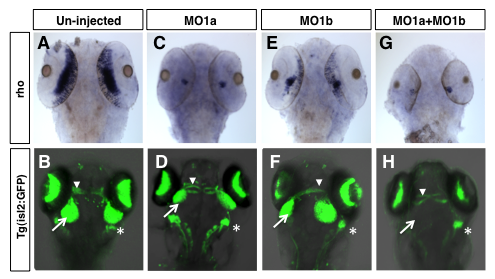

Supplement: Additional file 4 — Distinct patterns of rhodopsin expression and retinotectal projections in dmbx1a and dmbx1b morphant embryos. Dorsal view anterior to the top of un-injected (A, B), MO1a injected (C, D), MO1b injected (E, F), and MO1a + MO1b injected (G, H) embryos demonstrating expression of rhodopsin (rho) (A, C, E, G) or the retinotectal projection pattern (green fluorescence) as defined in isl2b:GFP transgenic embryos (B, D, F, H). The mismatch control injected morpholinos resembled the un-injected controls and are not shown. In B, D, F, H, white arrow demarcates the terminal field of the retinal ganglion cells in the optic tectum and the white arrowhead demarcates axonal fibers of the optic nerve. MO, morpholino. Asterisk demarcates the region of trigeminal ganglion. [file 1471-213X-10-100-S4.TIFF]

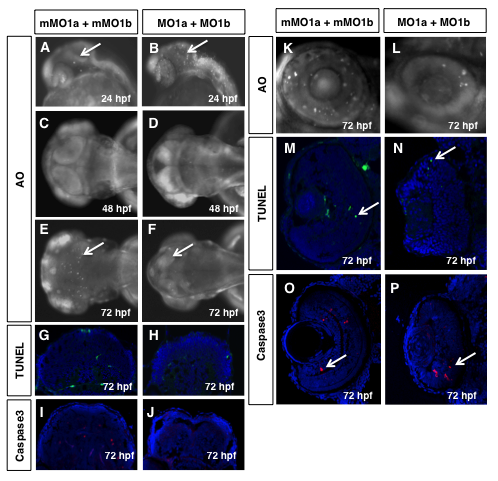

Supplement: Additional file 5 — Cell death does not persist in dmbx1 morphant embryos. Live embryos at 24 hpf (A, B, lateral view anterior to the left), 48 hpf (C, D, dorsal view anterior to the left) and 72 hpf (E, F, dorsal view anterior to the left; K, L, close up of retina lateral view dorsal to the top) were examined for the presence of apoptotic cells using AO. Arrows point to AO+ cells in the midbrain (bright spots). TUNEL+ cells (green) or Caspase3+ cells (red) on cryosectioned tissue of 72 hpf midbrains (G-J) and retina (M-P, arrow pointing to labelled cell) counter-stained with DAPI (blue). (MO, morpholino; mMO, mismatch morpholino; hpf, hours post-fertilization; AO, acridine orange. [file 1471-213X-10-100-S5.TIFF]

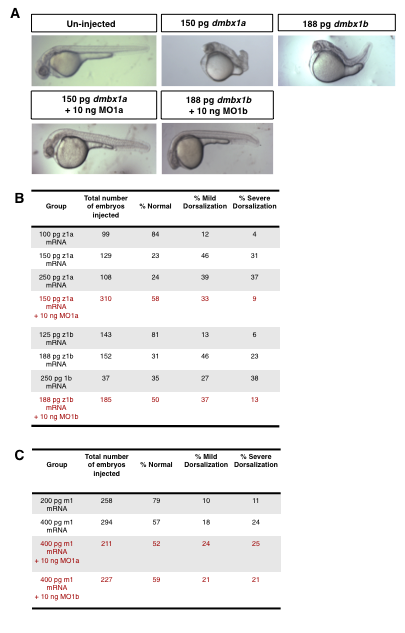

Supplement: Additional file 6 — Zebrafish, but not mouse, Dmbx1 mRNA counteracts the zebrafish knockdown phenotype. (A) Representative images of single embryos, lateral view anterior to the left from the different groups analyzed for a dorsalization phenotype. Embryos were injected with the mRNA concentrations shown, co-injected with 10 ng of either MO1a or MO1b as indicated, and scored for a dorsalized phenotype at 24 hpf. Tabulated results for all groups from 2-5 separate experiments using (B) zebrafish mRNA, or (C) mouse mRNA. MO, morpholino; hpf, hours post-fertilization. [file 1471-213X-10-100-S6.TIFF]

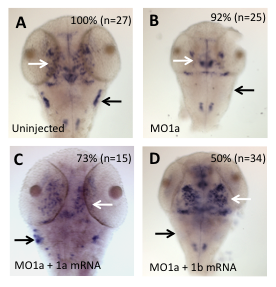

Supplement: Additional file 7 — Partial rescue of dmbx1a morphant with dmbx1b mRNA. (A) Un-injected and (B) MO1a injected embryos showing foxb1.2 expression at 48 hpf. Note, the data in these two panels are identical to panels (A) and (B) shown in Figure 9; they were duplicated here for ease of reference. Representative images of embryos co-injected with MO1a and either dmbx1a mRNA (C) or dmbx1b mRNA (D). Dorsal view of embryos, anterior to the top. Black arrows in all panels point to the anterolateral hindbrain region where foxb1.2 is normally expressed. White arrows in all panels point to the dorsal midbrain. MO, morpholino. [file 1471-213X-10-100-S7.TIFF]

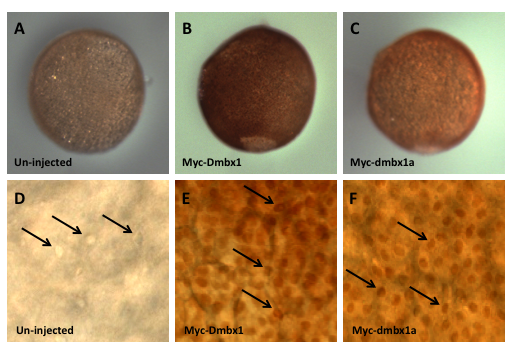

Supplement: Additional file 8 — Monitoring mouse Myc-Dmbx1 and zebrafish Myc-dmbx1a levels in vivo. Representative images of an (A) un-injected, (B) mouse myc-Dmbx1 mRNA injected (400 pg), and (C) zebrafish myc-dmbx1a mRNA injected (150 pg) tailbud stage embryo (lateral view) processed for whole-mount immunolabeling using an anti-Myc antibody (n = 10 per group). Panels D-F are representative high magnification images of embryos in each of the three treatment groups; black arrows indicate nuclei that are DAB+ in the injected groups, but lack DAB staining in the un-injected group. [file 1471-213X-10-100-S8.TIFF]

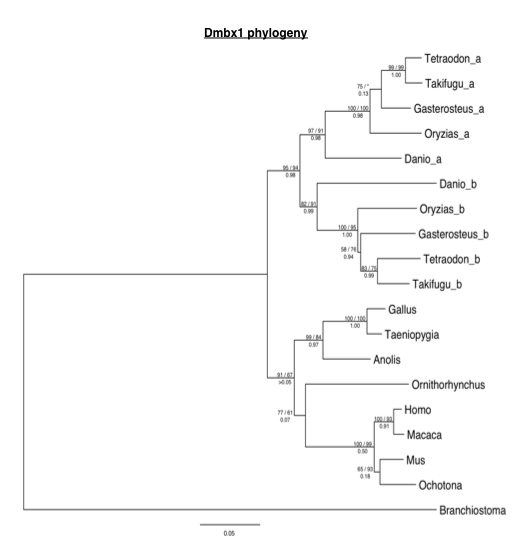

Supplement: Additional file 9 — Phylogenetic analysis of vertebrate Dmbx1 genes. Phylogram showing the neighbour-joining tree topology, with support values indicated for each node in the tree, for the three different phylogenetic methods of analysis employed. Above the nodes are neighbour-joining (1000 replicates), followed by likelihood bootstrap percentages (100 replicates); below the nodes are the Bayesian posterior probabilities. Bootstrap percentages below 50% are indicated by an asterisk. [file 1471-213X-10-100-S9.TIFF]

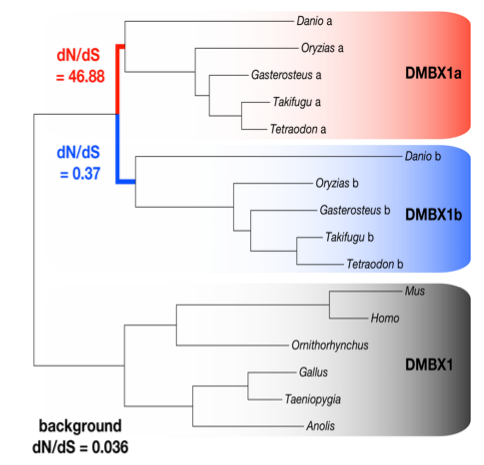

Supplement: Additional file 10 — Molecular evolutionary analysis of vertebrate Dmbx1 genes. Proportions of non-synonymous to synonymous rates (dN/dS) along lineages in the Dmbx1 phylogeny were estimated using codon-based maximum likelihood phylogenetic methods. Results of a branch model in which the post-duplication branches (PDBs) leading to the dmbx1a and dmbx1b clades were each allowed to have independently estimated dN/dS values are shown. Both PDBs display elevated dN/dS estimates compared to the background estimate applied to the remainder of the phylogeny. This model fit the data significantly better than a model with a single dN/dS parameter (M0 model; p < 0.01, d.f. = 2). Increases in dN/dS along each of the PDBs were also confirmed through simpler branch models in which only a single PDB, either for dmbx1a or dmbx1b, received separately estimated dN/dS values; in both cases the increases in dN/dS was statistically significant (M0; p < 0.05, d.f. = 1). Branch lengths shown in this figure were estimated under the 2 PDB branch model under which the dN/dS estimates were derived, and are proportional to the number of substitutions per codon. [file 1471-213X-10-100-S10.TIFF]
